# LightweightUNet: Multimodal Deep Learning with GAN-Augmented Imaging Data for Efficient Breast Cancer Detection

**DOI:** 10.3390/bioengineering12010073

**Published:** 2025-01-15

**Authors:** Hari Mohan Rai, Joon Yoo, Saurabh Agarwal, Neha Agarwal

**Affiliations:** 1 School of Computing, Gachon University, Seongnam 13120, Republic of Korea; drhmrai@gachon.ac.kr; 2 Department of Information and Communication Engineering, Yeungnam University, Gyeongsan 38541, Republic of Korea; 3 School of Chemical Engineering, Yeungnam University, Gyeongsan 38541, Republic of Korea

**Keywords:** LightweightUNet, breast cancer detection, multimodal approach, GAN-augmentation, synthetic dataset generation, deep learning, lightweight architecture

## Abstract

Breast cancer ranks as the second most prevalent cancer globally and is the most frequently diagnosed cancer among women; therefore, early, automated, and precise detection is essential. Most AI-based techniques for breast cancer detection are complex and have high computational costs. Hence, to overcome this challenge, we have presented the innovative LightweightUNet hybrid deep learning (DL) classifier for the accurate classification of breast cancer. The proposed model boasts a low computational cost due to its smaller number of layers in its architecture, and its adaptive nature stems from its use of depth-wise separable convolution. We have employed a multimodal approach to validate the model’s performance, using 13,000 images from two distinct modalities: mammogram imaging (MGI) and ultrasound imaging (USI). We collected the multimodal imaging datasets from seven different sources, including the benchmark datasets DDSM, MIAS, INbreast, BrEaST, BUSI, Thammasat, and HMSS. Since the datasets are from various sources, we have resized them to the uniform size of 256 × 256 pixels and normalized them using the Box-Cox transformation technique. Since the USI dataset is smaller, we have applied the StyleGAN3 model to generate 10,000 synthetic ultrasound images. In this work, we have performed two separate experiments: the first on a real dataset without augmentation and the second on a real + GAN-augmented dataset using our proposed method. During the experiments, we used a 5-fold cross-validation method, and our proposed model obtained good results on the real dataset (87.16% precision, 86.87% recall, 86.84% F1-score, and 86.87% accuracy) without adding any extra data. Similarly, the second experiment provides better performance on the real + GAN-augmented dataset (96.36% precision, 96.35% recall, 96.35% F1-score, and 96.35% accuracy). This multimodal approach, which utilizes LightweightUNet, enhances the performance by 9.20% in precision, 9.48% in recall, 9.51% in F1-score, and a 9.48% increase in accuracy on the combined dataset. The LightweightUNet model we proposed works very well thanks to a creative network design, adding fake images to the data, and a multimodal training method. These results show that the model has a lot of potential for use in clinical settings.

## 1. Introduction

Breast cancer accounts for over 11.6% of all cancer cases globally, making it the second most prevalent cancer category out of more than 200. It ranks first globally and is the most common cancer in women. Worldwide, over 6844 women lost their lives to breast cancer in 2020, with an estimated 2,300,000 new cases identified that year [1,2]. Metastatic (later) breast cancer has a much lower 5-year survival rate (15%) compared to early-stage (90%) diagnoses [3]. The diagnosis of breast cancer with a traditional approach requires a physical examination by a radiologist or medical experts; this may be an invasive or non-invasive method. However, non-invasive techniques are preferred for automatic and computerized detection, especially when using the AI approach. Medical images of breasts of various modalities, including mammogram, CT, Ultrasound, MRI, and histopathology are the best examples of non-invasive techniques [4]. Among these many modalities, we have utilized mammography (MGI) and ultrasound (USI), where MGI is low dose-Xray imaging which captures the detailed structure of the affected area [5,6]. However, the limitation of MGI is that it may cause an ionizing radiation effect, which has a small impact on secondary cancers. It has also not been very effective in distinguishing grades of breast cancers such as benign and malignant [7]. Because of these limitations, another non-invasive and effective alternative to the MGI modality is USI, which utilizes high-frequency sound waves to produce real-time images of the breast tissues [8]. It provides enhanced and detailed information about benign and malignant cancer [9]. This is the reason we have combined both modalities, MGI and USI, for the multimodal approach in this work. This manuscript proposes a novel methodology that bridges this gap by utilizing the combined powers of both USI and MGI for improved breast cancer detection.

Understanding the complexities of breast cancer is crucial to identifying the favorable outcomes of our proposed method. Breast cancer is triggered by the abnormal development of cells inside breast tissue, and its degree of seriousness differs according to the category and stage of the disease. Early-stage tumors are called benign, which are not spreadable or non-cancerous and can be treated easily when detected early. Benign cancer is less dangerous and is diagnosed in around one in every eight women over their lifetime and is often limited. Malignant tumors, on the other hand, can spread (metastasize) to different regions of the body if not diagnosed early [1,10]. Malignant tumors are cancerous, and fatality is very high among them, with a lower survival rate.

Breast cancer is triggered by a variety of risk factors, which include aging (as risk rises with age), family history (having a close relative with the disease increases risk), genetic mutations (BRCA1 and BRCA2 gene mutations significantly raise risk), hormonal imbalances (long-term exposure to estrogen can increase risk), and eating and lifestyle habits (obesity and extreme alcohol drinking are associated with higher risk) [11,12]. Breast cancer patients have a significantly improved survival rate when the disorder is recognized at an initial phase because of extensive screening methods.

A multimodal strategy, which incorporates information from both USI and MGI, has unique advantages over relying on just a single modality [4,13]. The proposed method attempts to improve breast cancer detection rates by combining the features of both modalities. Mammography is particularly good at identifying microcalcifications, which are frequently associated with early-stage malignancies, whereas ultrasonography can successfully distinguish between solid and fluid-filled lesions. The use of this information is that a combination of the two modalities will give better results that are more accurate and reliable. Factually, it is evident that “mammography may generate false positives, causing anxiety for those having to undergo treatments”. Ultrasounds do not have all the necessary capabilities to determine lesion features, which will definitely help to reduce false positives. Still, ultrasound is a better source to assess whether tumors are present and vital for breast cancer cases. In general, mammography is a more detailed source that provides a more complex level of structural information, whereas ultrasonography is better for soft tissue vision.

In this context, our proposal of a multimodal method, a combination of both modalities, provides better accuracy and a more reliable breast cancer diagnosis. By blending the strengths of both modalities, we find a method to overcome the limitations of each approach. The LightweightUNet model, designed for effectively detecting breast cancer, has a low computational cost, a smaller number of layers, and is capable of running on portable devices, too. This approach could reform breast cancer screening with access to modern healthcare facilities. This has the potential to significantly enhance detection rates and accelerate early diagnosis, ultimately leading to better patient outcomes.

The key contributions and highlights of this work are as follows:

- A novel LightweightUNet deep learning model for effectively detecting breast cancer from large imaging datasets.
- The proposed model’s key feature is its low computational cost. With only nine layers, it is compact and requires significantly less processing power than complex DL models.
- The LightweightUNet model uses a multimodal approach to training. It utilizes 13,000 images from two different modalities, mammograms, and ultrasound, collected from various sources.
- Seven benchmarked databases were utilized: DDSM, MIAS, INbreast, BrEaST, BUSI, Thammasat, and HMSS, containing benign and malignant pathology types, providing the foundation for the early detection of breast cancer.
- Synthetic data generation was utilized to solve the limitation of adequate data for training the DL models. The StyleGAN3 model was used to generate 10,000 synthetic USI images.
- The proposed model was trained using a large dataset containing 20,400 images of two modalities (MGI, USI).
- The impact of high-quality synthetic images on the performance of the proposed model was investigated using a 5-fold cross-validation technique. The model was validated on two datasets: a real dataset without augmentation and a combined dataset including real and synthetic images generated using a generalized adversarial network (GAN).
- Over a 9% significant improvement in key performance metrics was observed in the performance of the proposed LightweightUNet model on the combined dataset (Real + GAN).

The manuscript’s remaining sections are organized as follows: Section 2 thoroughly describes the materials, methods, and proposed LightweightUNet model design, including its key improvements. Section 3 describes the experimental setup, training methods, data augmentation strategies, and classification results. Section 4 provides a discussion that compares LightweightUNet with previous approaches. Finally, Section 5 summarizes major findings, emphasizes the model’s relevance, and discusses potential future research possibilities.

## 2. Literature Review

Breast cancer remains a leading cause of death among women globally; early detection plays a crucial role in improving patient outcomes. In recent years, advancements in deep learning and machine learning have revolutionized the field of breast cancer detection. This literature review examines the innovative approaches employed in recent studies, focusing on the features and techniques utilized, the datasets leveraged, and the achieved accuracy metrics (Table 1).

As shown in Table 1, various methodologies have been explored for breast cancer detection. While deep learning approaches, particularly convolutional neural networks (CNNs), have achieved impressive accuracy rates (e.g., Khuriwal et al. [14] at 98.00%, Zhao et al. [20] at 98.09%), AlSalman et al. [29] at 98.9%). Existing research often focuses on single modalities, such as mammography or ultrasound (e.g., Bouzar-Benlabiod et al. [25], Aidossov et al. [23]). While these modalities offer valuable insights, they may have limitations in capturing the full picture of breast lesions. Combining information from multiple modalities (e.g., Oyelade et al. [30]) has the potential to improve detection accuracy, but effective techniques for data fusion and feature extraction remain a challenge. Accuracy metrics reported in the literature vary considerably (e.g., Khuriwal et al. [14] at 98.00% vs. Bouzar-Benlabiod et al. [25] at 86.71%). This variability can be attributed to differences in dataset size, composition, and the specific challenges each study addresses. Establishing standardized benchmarks and addressing dataset limitations are crucial for ensuring robust and generalizable models. Many research works (e.g., Zhao et al. [20], Asadi and Memon [26]) lack assessment of external datasets, which generates concerns regarding the generalizability of their outcomes to real-world clinical scenarios. Rigorous validation approaches have to be followed to ensure a model’s performance in a variety of clinical contexts. For some CNN models (e.g., Sait and Nagaraj [27]), interpretability is still a challenge, comparable to other deep learning models. For greater confidence and clinical adoption, clinicians need models that offer perspectives on their decision-making processes.

In recent years (2022–2025), many researchers have developed lightweight deep learning (DL) models to address the challenges of breast cancer detection, particularly in resource-constrained environments (Laxmisagar et al. [32] used Lightweight DNN techniques, including the MobileNet CNN with categorical cross-entropy, achieving 87.5% accuracy. However, it requires considerable computing power, including GPUs, which restricts its use in portable devices in resource-constrained environments. Nneji et al. [33] presented the Lightweight Separable Convolution (LWSC) model and provided high performance with 97.23% accuracy, 97.71%, and 97.93% sensitivity and specificity, respectively. However, further validation of more diverse datasets is required. Yan et al. [34] proposed the Privacy-Embedded Lightweight and Efficient Automated (PLA) framework, which provided high performance with classification accuracy, recall, f1, and precision of 95.3%, 99.8%, 96.9%, and 98.8%, respectively. The methodology included the privacy-preserving techniques but requires additional validation in real-world IoMT scenarios. Kausar et al. [35] presented lightweight techniques for breast cancer detection by integrating Wavelet Transform (WT) with lightweight CNN and Invertible Residual Blocks, achieving an accuracy of 96.25% on the ICIAR 2018 dataset and 99.8% on BreakHis. However, performance on the Bracs dataset dropped to 72.2%. Further validation of diverse datasets is necessary. Oladimeji et al. [36] employed a lightweight model that incorporates entropy-based slice selection techniques, achieving a 91% accuracy rate while reducing computational complexity. The model, however, requires validation of larger and more varied datasets. Lightweight models are employed in various medical imaging tasks like skin lesion detection and segmentation [41,42,43], while LightCF-Net [44] is applied to polyp segmentation.

Elaraby et al. [37] presented a lightweight CNN (LWCNN) model for the detection of breast cancer utilizing mammogram images, achieving training and testing accuracy ranging from 95% to 99%. Further evaluation of a wider dataset and analysis of false positives and negatives are needed. Saha et al. [38] introduced the lightweight DCNN model Breast-NET, utilizing transfer learning. The model achieved the highest accuracy of 90.34%, precision of 0.86, recall of 0.92, and F1 score of 0.89. Despite promising results, the model’s validation was limited to specific datasets, with concerns about resource requirements for deployment. Nguyen Chi et al. [39] also presented the combined ResNet34 with Chi-Square Filter and SVM for breast cancer detection. The methodology provided the highest accuracy of 99.62%, with an 18.3% improvement over standard CNN models. While highly effective, this approach needs further evaluation for generalizability and requires considerable computational resources for training.

Tang et al. [40] developed the Multi-light Net (lightweight model), enhanced detection accuracy for breast cancer detection with thermal imaging, and Weighted Label Smoothing Regularization (WLSR) for improved generalization and stability. However, its evaluation on mobile devices was limited, necessitating more extensive testing.

The findings suggest that lightweight models are becoming better at diagnosing breast cancer; however, they still need to be verified on larger and multimodal datasets, in more real-world circumstances, with improved generalizability and efficiency before they can be widely implemented.

These limitations in the literature emphasize the need for novel approaches to bridge the disparity between accurate results, practical application, and expanded clinical significance. In order to overcome these drawbacks, our proposed LightweightUNet model provides a number of benefits over the approaches described in the review table. It is possible to modify LightweightUNet to integrate information from several imaging modalities, which might result in more accurate detection. Because LightweightUNet optimizes computational efficiency, it can be easily deployed on portable devices and makes point-of-care screening more accessible, especially in situations with limited resources. In order to promote mutual confidence and comprehension among healthcare professionals, we developed our model with interpretability in mind. LightweightUNet has the potential to make a substantial contribution to the development of breast cancer detection technologies by filling in these research gaps.

## 3. Materials and Methods

**Figure 1:**
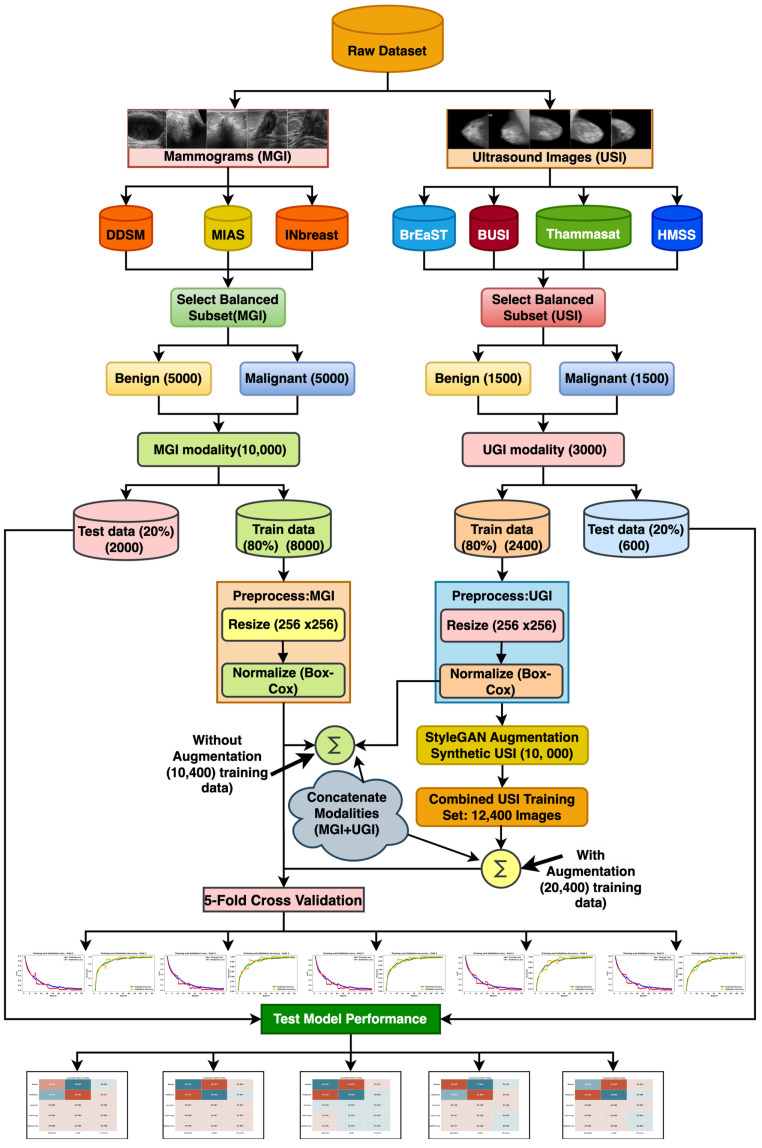
LightweightUNet-driven breast cancer detection methodology.

This section outlines the materials and techniques employed in the development of the novel LightweightUNet model for breast cancer detection. Figure 1 illustrates the overall methodology employed, offering a visual representation of the complete process. The first stage involves gathering and curating a large-scale dataset on breast cancer. Next, preprocessing methods are applied to the data to ensure both consistency and quality. Then, to address any data limitations and improve the training dataset, data augmentation techniques were put into place. Subsequently, an in-depth description of the LightweightUNet model architecture is provided, emphasizing its basic elements and innovative methods that enhance its effectiveness and accuracy. Finally, the specific assessment measures are used to carefully evaluate how well the algorithm classified cases of breast cancer.

### 3.1. Multimodal Datasets

The two main imaging modalities used in our research are mammograms (MGI) and ultrasound images (USI), and we employed various open-source datasets for detecting breast cancer. We have collected the datasets from 7 different sources, with 3 for MGI and 4 for USI. Table 2 provides details about the utilized datasets and their pathology types from all seven sources. In MGI modalities, we have utilized three benchmark datasets: a digital database for screening mammography (DDSM) [45], Mammographic Image Analysis Society (MIAS) [46], and INbreast [47]. The DDSM dataset contains the highest number of images, 10,239, whereas the MIAS dataset and INbreast dataset contain fewer images, 322 and 410, respectively. To maintain the uniformity of both pathology types (benign and malignant), we have selected an equal number of images, 5000 each, from both pathology types, totaling 10,000 images from MGI modalities.

From USI modalities, we have used four datasets from 4 different sources: BrEaST, BUSI, Thammasat, and HMSS. These datasets contain varying numbers of images and pathology types: 256 images in the BrEaST dataset [48], 780 images in the BUSI dataset [49], 263 images in the Thammasat dataset [50], and the highest, 2006 images, in the HMSS dataset [51]. To reduce the possibility of data discrepancies that may distort our research, we concentrated on the two primary pathology categories: benign and malignant. For our first investigation stage, we utilized 3000 ultrasound images overall, having once again chosen a balanced selection of 1500 images from every type.

**Figure 2:**
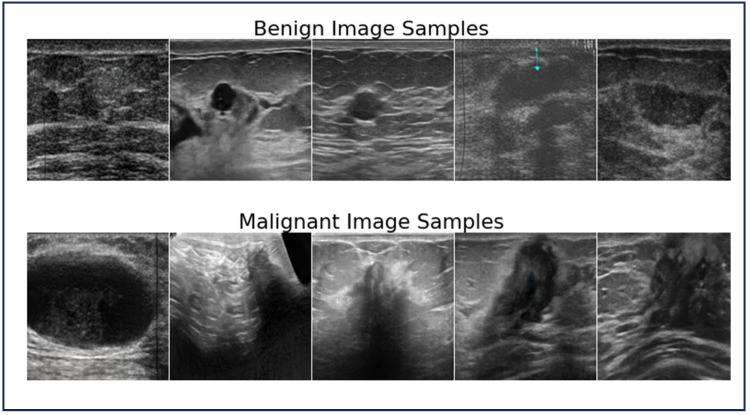
Sample images of benign and malignant pathology types from USI modality.

**Figure 3:**
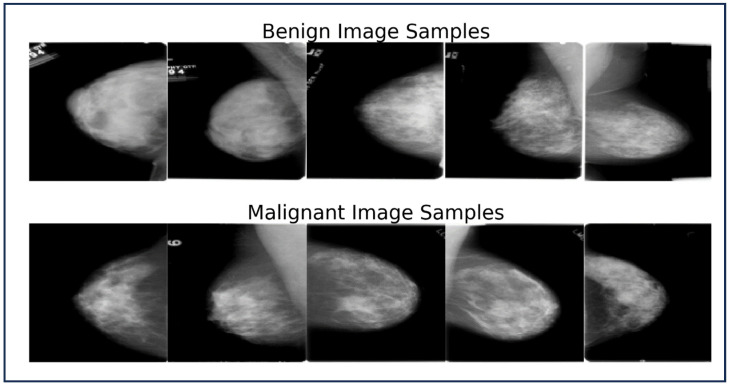
Sample images of benign and malignant pathology types from MGI modality.

To sum up, a total of 13,000 images were used in our initial research; 10,000 images were from the MGI modality, and 3000 images were obtained from USI. In order to provide an in-depth examination of breast cancer detection methods, this balanced selection of datasets provides a fair representation of both benign and malignant cases. To aid understanding and visualization of the different pathology types, representative images from both modalities (USI and MGI) are presented. Figure 2 and Figure 3 showcase the samples of benign and malignant lesions from USI and MGI datasets, respectively.

### 3.2. Data Preprocessing

In the field of medical image analysis, preprocessing is an essential preliminary phase, particularly when dealing with data obtained from multiple sources and imaging modalities. We explored two modalities, MGI and USI, in the utilized datasets collected from seven different sources [6,7].

Our methodology begins by addressing the typical difference between the sizes of images within datasets. Since our research utilizes images from various modalities and sources, preserving uniformity in image size is crucial for further evaluation. We have carefully chosen the typical size of 256 × 256 pixels, which ensures uniformity across all images. We employed a standardization approach on each image in each dataset to achieve this uniformity. During this laborious process, each image is iteratively resized to specified dimensions using an appropriate algorithm. This rigorous process ensures that the image sizes are identical across all datasets, enabling accurate evaluations and assessments.

In the second step of our preprocessing procedures, we used the Box-Cox Transformation normalizing technique. Before feeding data as input to the machine learning models, normalization is an important step in the preprocessing process that helps ensure uniformity and eliminate any biases [52]. Biases can arise from multiple sources, such as distinct imaging modalities (MGI and USI in our case) or slightly different settings on the acquisition devices. By transforming the data into an acceptable range or distribution, the normalization procedure contributes to establishing balanced instances for analysis [53,54].

For normalization, the Box-Cox transformation method was specifically selected because of its unique ability to handle cases when the distribution of pixel intensities within the image does not follow a normal distribution (a bell-shaped curve). Many image datasets have non-normal distributions, and Box-Cox Transformation is efficient at addressing this issue. The Box-Cox transformation can be expressed mathematically using Equation (1) [53].

(1) $$X^{\lambda }=\left\{\begin{array}{c} \frac{X^{\lambda }-1}{\lambda },\;for\;\lambda \neq 0\\ log\;(X),\;for\;\lambda =0 \end{array}\right.$$

where X is the original dataset and λ is the transformation parameter. Using Box-Cox Transformation, we normalized the pixel intensities of every image in the MGI and USI datasets.

Algorithm 1 describes the preprocessing steps applied to the multimodal breast imaging dataset, including MGI and USI. In the first step, we applied the image size standardization, where each image is resized to a uniform resolution $\left({\mathrm{width}}_{\mathrm{out}},{\mathrm{height}}_{\mathrm{out}}\right)$ to ensure consistency and compatibility. Then, we applied the proposed Box-Cox normalization to stabilize the image intensity distributions and approximate a Gaussian distribution, which enhances the numerical stability during training. Finally, the output of the preprocessed algorithm provided standardized and normalized pixel intensity, ensuring improved image quality and consistency for accurate breast cancer detection using the proposed techniques.


**Algorithm 1:** Preprocessing techniques for the multimodal breast images**Input:** Datasets (D)$({D_{\mathrm{MGI}},\;D}_{\mathrm{USI}}$)
**# Step 1: Image size standardization:**
**for** each dataset (${D_{\mathrm{MGI}},\;D}_{\mathrm{USI}}$) in D:  **for** each image ($I_{i}$) in the dataset:   I_i_prime = Resize ($I_{i},width_{out},height_{out}$)
**# Step 2: Normalization using Box-Cox transformation**
**for** each dataset ($D_{MGI},D_{USI}$) in D:  **for** each image ($I_{i}$) in the dataset:    transformed_image = apply_BoxCoxTransformation $\left(I_{i}\right)$
**def** apply_BoxCoxTransformation(image):  λ = 0.5      **if** λ ! = 0:      
$\mathrm{transformed}\text{\_}\mathrm{image}=\frac{{\mathrm{image}}^{\lambda }-1}{\lambda }$
    
**else:**
      
$\mathrm{transformed}\text{\_}\mathrm{image}=\log\left(\mathrm{image}\right)$
     **return** transformed_image


### 3.3. Synthetic Image Generation Using StyleGAN3

**Figure 4:**
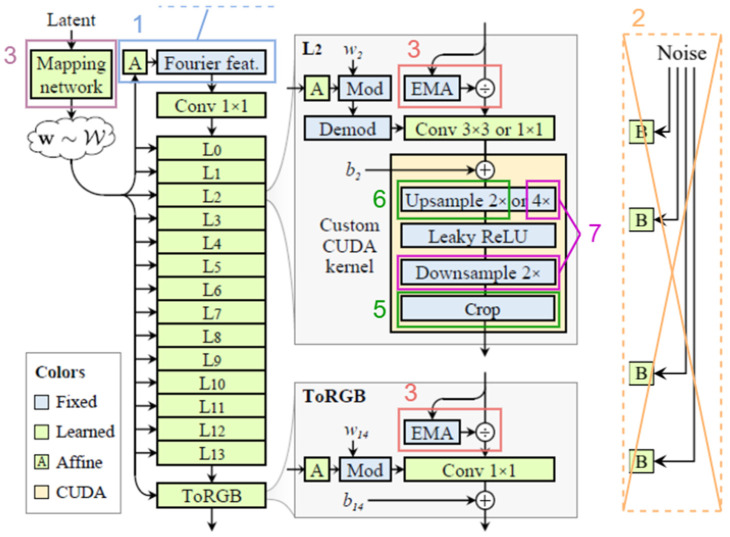
The structural view of the StyleGAN3 model [56].

The datasets utilized in this study are of two different modalities: mammography and ultrasound. There are 3000 ultrasound images and 10,000 mammogram images in the utilized dataset, which raises the data imbalance issue between the two modalities. Due to the comparatively small number of images in the ultrasound dataset, it may not be adequate to train the model properly. So, we tackled this data imbalance problem between both modalities, MGI and USI, utilizing StyleGAN3 [55]. StyleGAN3 is an extended version of the generalized adversarial network (GAN) designed for high-fidelity image synthesis. It also contains a generator and discriminator, where the generator generates the synthesized images, and the discriminator differentiates between real and fake images. Figure 4 presents the layer-wise structure of the StyleGAN3 model, which is utilized for the generation of high-quality ultrasound images.

It has a mapping network (*Gm*), which is an 8-layer multilayer perceptron (MLP) used to transform the latent noise vector (*z*) into the intermediate latent space (*W*). Equation (2) defines the style vector (*w*), which controls the output of the generator network.

(2) $$w=G_{m}\left(z\right)=\phi \left(W_{n}\phi \left(W_{n-1}\ldots \phi \left(W_{2}\phi \left(W_{1}z+b_{1}\right)+b_{2}\right)\ldots +b_{n-1}\right)+b_{n}\right)$$

The style vector (*w*) in styleGAN3 modulates the weight of each layer of the synthesis network (*Gs*) using adaptive instance normalization (AdaIN), which is expressed mathematically by Equation (3).

(3) $$\mathrm{AdaIN}\left(x,y_{s},y_{b}\right)=y_{s}\left(\frac{x-\mu \left(x\right)}{\sigma \left(x\right)}\right)+y_{b}$$

where, $y_{s},y_{b},\mu \left(x\right),\sigma \left(x\right)$ are style parameters, bias parameters, mean of feature map, and standard deviation of the feature map, respectively. To control the feature map, the style vector (*w*) is incorporated into each layer through the synthesis network (*G_s_*), which is mathematically represented by Equation (4).

(4) $$G_{s}\left(w\right)=\sum_{l}\mathrm{AdaIN}\left(U\left(W_{l}*F_{l}\right),y_{s,l},y_{b,l}\right)$$

where, $U,W_{l},F_{l},y_{s,l},y_{b,l}$ are upsampling operation, weight matrix of layer “*l*”, layer “*l*” feature map, style scale, and bias parameters derived from weight w at layer *l*.

The StyleGAN3 also utilizes a progressive, growing technique, in which both the network discriminator and generator begin with low-resolution images and gradually increase the resolution by adding the number of layers, as $\mathrm{Res}\left(l\right)=2^{l}\times 2^{l}$. It also presents the alias-free design to prevent visual artifacts, which prevents the injection of high-frequency artifacts by specifically and carefully designing the downsampling and upsampling operations. This ensures that the transformation follows the Nyquist sampling theorem, preserving signal integrity in the styleGAN3 model. It also utilizes the Fourier Transform (FT) features to further improve the generation quality, which enables the model to extract fine details and global structure more effectively. The modulated feature map using the Fourier transform is expressed by Equation (5).

(5) $$\begin{array}{c} F_{\mathrm{modulated}}\left(x,y\right)=\frac{1}{MN}\sum_{u=0}^{M-1}\sum_{v=0}^{N-1}[\left(\sum_{x^{\prime }=0}^{M-1}\sum_{y^{\prime }=0}^{N-1}F\left(x^{\prime },y^{\prime }\right)e^{-2\pi i\left(\frac{ux^{\prime }}{M}+\frac{vy^{\prime }}{N}\right)}\right)\\ \cdot \left(\sum_{x^{\prime \prime }=0}^{M-1}\sum_{y^{\prime \prime }=0}^{N-1}\mathrm{Mod}\left(x^{\prime \prime },y^{\prime \prime }\right)e^{-2\pi i\left(\frac{ux^{\prime \prime }}{M}+\frac{vy^{\prime \prime }}{N}\right)}\right)]e^{2\pi i\left(\frac{ux}{M}+\frac{vy}{N}\right)} \end{array}$$

In our proposed methodology, we have used the preprocessed images to train the styleGAN3 model to generate synthetic images. We have used ultrasound images to train and generate synthetic images, with training images (2400, i.e., 80% of the total 3000), and the remaining 600 are kept reserved for testing the proposed model. As the StyleGAN3 model is capable of generating high-quality images, we have decided to generate 256 × 256 images. This choice reduces the training time of the proposed model while maintaining the adequate quality of the images. Very high-resolution images take a longer duration to train the models and increase the computational cost, whereas very low-resolution images may fail to extract the appropriate features and result in poor classification outcomes, especially in medical imaging.

Data is key to training any model effectively. Since we did not have a lot of ultrasound (USI) data initially, we used a technique called StyleGAN3 to create 10,000 high-resolution (256 × 256 pixels) synthetic USI images. This significantly boosted our dataset and allowed us to train a strong classification model. We trained the StyleGAN3 model autonomously for every single lesion in order to ensure that the generated images accurately represent the unique types of lesions present in the real dataset. By employing this approach, the model was able to acquire the unique features of each type of lesion, leading to the generation of high-resolution synthetic images that showed characteristics unique to each category.

We used an extremely rigorous training approach to make sure the StyleGAN3 model fully learned all the complex patterns in the USI dataset. To be more specific, we applied the “—kimg = 1000” parameter, enabling the model to process one million real USI images prior to training completion. The model was able to produce synthetic images that are very identical to real medical images due to this extended visibility, which allowed it to represent the tiny variances found in real ultrasound data accurately. The Frechet Inception Distance (FID) is a metric that assesses the similarity between generated synthetic images and actual USI data, serving as a tool for evaluating the quality of these images. A lower FID score indicates greater similarity and, consequently, higher image quality. The FID measure is mathematically defined by Equation (6).

(6) $$\mathrm{FID}=\left.|\mu_{r}-\mu_{g}\right.{|}^{2}+\mathrm{Tr}\left(\Sigma_{r}+\Sigma_{g}-2{\left(\Sigma_{r}\Sigma_{g}\right)}^{1/2}\right)$$

where Tr is the trace of a matrix, $\Sigma_{r}$ and $\Sigma_{g}$ are the covariance matrices of real and generated images, and $\mu_{r}$ and $\mu_{g}$ are the means. In our case, the malignant lesions had an FID score of 22.9 and the benign lesions had an FID score of 35.4. These results demonstrate how well-produced and authentic the synthetic images generated by StyleGAN3 are at the preferred 256 × 256-pixel resolution.

#### Ultrasound Images Generation

Our method uses random noise vectors to create realistic ultrasound images by utilizing the StyleGAN3 architecture. High-resolution ultrasound images, denoted as x, are generated by the GAN generator model, which maps these latent space vectors $\left[z∼N\left(0,1\right)\right]$ and incorporates random noise vectors to produce fine details. Convolution blocks that are layered from the generator’s core [57] gradually improve the produced images’ resolution. Equation (7) shows the key components that enable the generator to acquire both fine-grained features and larger-scale information.

(7) $$x_{i}=G\left(z_{i}\right)\;\mathrm{where}\;z_{i}∼N\left(0,I\right)$$

The component of StyleGAN3, known as the discriminator, is responsible for distinguishing between actual $\left(x_{\mathrm{real}}\right)$ and producing $\left(x_{\mathrm{real}}\right)$ ultrasound images. In order to do this, it employs a sequence of convolutional blocks, which are represented by Equation (8).

(8) $$\begin{array}{c} P_{\mathrm{real}}=D\left(x_{\mathrm{real}}\right)\\ P_{\mathrm{fake}}=D\left(x_{\mathrm{fake}}\right) \end{array}$$

The output of the discriminator is represented by *D*(*x*), which is a probability score ranging from 0 to 1 that represents the likelihood that *x* is real. The discriminator’s loss function is calculated using this score in the classification scenario by binary (real or fake). Equations (9)–(11) provide the individual loss functions for the real data ($L_{D,\mathrm{ra}})$, fake data ${(L}_{D,\mathrm{fk}})$, and the total discriminator loss ($L_{D})$, respectively.

(9) $$L_{D,\mathrm{ra}}=-E_{x_{\mathrm{ra}}∼p_{\mathrm{dt}}\left(x\right)}\left[\log\left(\frac{1}{1+\exp\left(-D\left(x_{\mathrm{real}}\right)\right)}\right)\right]$$

(10) $$L_{D,\mathrm{fk}}=-E_{z∼N\left(0,I\right)}\left[\log\left(1-\frac{1}{1+\exp\left(-D\left(G\left(z\right)\right)\right)}\right)\right]$$

(11) $$L_{D}=-E_{x_{\mathrm{ra}}∼p_{\mathrm{dt}}\left(x\right)}\left[\log\left(\frac{1}{1+\exp\left(-D\left(x_{\mathrm{real}}\right)\right)}\right)\right]\;\;\;\;\;\;\;\;\;\;\;\;\;-E_{z∼N\left(0,I\right)}\left[\log\left(1-\frac{1}{1+\exp\left(-D\left(G\left(z\right)\right)\right)}\right)\right]$$

The discriminator’s output, $P_{\mathrm{real}}\;\mathrm{and}\;P_{\mathrm{fake}},$ estimates the likelihood that each provided sample is genuine (real) or generated (fake), respectively, represented by Equations (12) and (13).

(12) $${\hat{y}}_{\mathrm{Real}}=\frac{1}{1+\exp\left(-P_{\mathrm{real}}\right)}$$

(13) $${\hat{y}}_{\mathrm{fake}}=\frac{1}{1+\exp\left(-P_{\mathrm{fake}}\right)}$$

A feature-matching loss $\left(L_{FM}\right)$ has been utilized to stabilize training and enhance the quality of images. Based on the variations in the statistics of features taken from created and real images, this loss imposes penalties on the generator. Equation (14) provides a mathematical definition of the feature-matching loss $\left(L_{FM}\right)$ for a given feature extractor, φ.

(14) $$L_{FM}=E_{z∼N\left(0,I\right)}|E_{x_{\mathrm{ra}}∼p_{\mathrm{dt}}\left(x\right)}\left[\phi \left(x_{\mathrm{real}}\right)\right]-E_{x_{\mathrm{fk}}∼p_{\mathrm{gn}\left(x\right)}\left[\phi \left(x_{\mathrm{fake}}\right)\right]\overset{2}{\underset{2}{|}}}$$

As indicated by Equation (15), a gradient penalty $\left(L_{GP}\right)$ is imposed to guarantee the discriminator fulfills the Lipschitz requirement.

(15) $$L_{GP}=E_{\hat{x}∼p_{\hat{x}}}\left[{\left(|\nabla_{\hat{x}}D\left(\hat{x}\right){|}_{2}-1\right)}^{2}\right]$$

where the uniform sampling of $\left(\hat{x}\right)$ is carried out from the images that are interpolated linearly between produced and actual images. StyleGAN3 gradually improves its ability to produce ultrasound images through the adversarial training process between the discriminator and generator. The generated images exhibit an exceptional degree of realism, precisely matching the attributes of genuine ultrasound data. This development provides an important tool for a range of medical imaging and research applications.

The distribution of our dataset across modalities (MGI and USI) and for training and testing purposes is presented in Table 3, both before and after applying StyleGAN augmentation. It is important to note that we consistently reserved 20% of the data from each modality (2000 images for MGI and 600 images for USI) for testing purposes. This 20% split remains unchanged throughout the process, as test data is typically not involved in augmentation or training. The key difference lies in the training data, particularly in the USI modality. Initially, the USI training set comprised 80% of its total data (2400 images). This portion of the USI data was then subjected to StyleGAN augmentation. This process successfully generated an additional 10,000 synthetic USI images, significantly bolstering the USI training data. Consequently, the total USI training set grew to 12,400 images after augmentation. In contrast, the MGI modality already possessed a sufficient amount of data (8000 images) for training. Therefore, it was not included in the StyleGAN augmentation process, and the training data remained at 8000 images for MGI. By leveraging StyleGAN, we effectively addressed the initial imbalance in the training data between the two modalities. The USI training data was significantly expanded (from 2400 to 12,400 images) through synthetic image generation, while the MGI training data size remained unchanged. This approach can potentially improve the performance of a machine learning model trained on this dataset by mitigating potential biases caused by data size discrepancies.

### 3.4. Depthwise Separable Convolution (DSConv)

Depthwise Separable Convolutions (DSConv) are a game-changer for convolutional neural networks (CNNs) deployed on mobile devices or environments with limited resources [58,59]. While CNNs excel at image recognition and other tasks, their reliance on standard convolution operations can be computationally expensive, especially when dealing with models designed for mobile platforms. DSConv addresses this challenge head-on by breaking down the standard convolution into two efficient steps, leading to significant improvements in processing speed. With this simplified approach, spatial information is firstly captured in each channel using a Depthwise Convolution block, and then it is combined across channels using a Pointwise Convolution block. ReLU activation and batch normalization both help to maximize network performance. The Depthwise and Pointwise Convolution blocks are the foundation of DSConv. Hence, it is crucial to highlight that understanding them fully requires a closer examination of their functions.

#### 3.4.1. Depthwise Convolution

The first step in DSConv, Depthwise convolution, addresses efficiency by concentrating on certain channels inside the input feature map. Depthwise convolution makes use of a collection of customized filters, each of which operates on a different input channel, as opposed to normal convolution, which applies a single filter to all channels [60]. Every channel is assigned a certain filter, usually a 3 × 3 size. Convolutional kernels, the mathematical notation for these filters, may be thought of as specialized tools made to extract spatial information unique to the data inside the channel; they are allotted $(c\in \left[1,C_{\mathrm{in}}\right],\;\mathrm{where}\;C_{\mathrm{in}}$ is the total number of input channels). This meticulous analysis is remarkably efficient. By using separate filters ($K_{i,j,c}$ in Equation (16)), depthwise convolution extracts features from each channel without inflating the overall number of channels in the output. The output feature map retains the same number of channels as the input (represented by the tensor shape ($H\times W\times C_{\mathrm{in}}$), where H is the height and W is the width). In essence, depthwise convolution acts like a team of analysts, each focusing on a specific channel with a dedicated tool. They efficiently extract crucial spatial information without creating additional channels, paving the way for the next stage of DSConv where the team’s findings are combined [60].

(16) $$Y_{h,w,c}=\sum_{i=0}^{K-1}\sum_{j=0}^{K-1}X_{h+i,w+j,c}\cdot K_{i,j,c}$$

where K is the kernel size and $K_{i,j,c}$ is the depthwise convolution filter for channel c.

#### 3.4.2. Pointwise Convolution (PWConv)

The second act of DSConv, pointwise convolution (PWConv), takes center stage. Unlike Depthwise convolution’s focus on individual channels, PWConv brings the team’s findings together. Imagine a single, small filter (typically 1 × 1) tasked with combining the outputs generated by each channel in the Depthwise convolution stage. This filter acts like a conductor, orchestrating a “linear combination” of the features extracted from each channel [61]. By combining these channel-specific features, PWConv allows the network to learn complex relationships between them. This enables the network to go beyond simply understanding individual channels and grasp the bigger picture—how these channels interact and contribute to the overall representation. Mathematically, Equation (17) represents this pointwise convolution operation. While the specifics might not be crucial at this point, it essentially translates the conductor’s instructions (represented by the filter) into a combined output feature map. In essence, PWConv leverages a small filter to combine the wealth of information extracted by the Depthwise convolution specialists. This collaboration unlocks the power of understanding not just individual channels but also the intricate interplay between them, leading to a more comprehensive representation [62].

(17) $$Z_{h,w,k}=\sum_{c=1}^{C_{\mathrm{in}}}Y_{h,w,c}\cdot P_{c,k}$$

where P is the pointwise convolution filter of shape $\left(C_{\mathrm{in}}\times C_{\mathrm{out}}\right)$ and Z is the final output tensor of shape $\left(H\times W\times C_{\mathrm{out}}\right).$ Putting it all together, the depthwise separable convolution combines these two steps, as given by Equation (18). $Z=\mathrm{PointwiseConvolution}\left(\mathrm{DepthwiseConvolution}\left(X\right)\right)$.

(18) $$Z=\sum_{c=1}^{C_{\mathrm{in}}}\left(\sum_{i=0}^{K-1}\sum_{j=0}^{K-1}X_{h+i,w+j,c}\cdot K_{i,j,c}\right)\cdot P_{c,k}$$

DSConv is revolutionizing resource-constrained CNNs. By separating standard convolution into efficient Depthwise and pointwise operations, DSConv drastically reduces computational costs, making it perfect for mobile devices and real-time applications. Despite this efficiency focus, DSConv maintains feature extraction through its two-stage approach: capturing spatial information in each channel (Depthwise) and then combining that information to learn complex relationships (pointwise). This allows DSConv models to achieve good performance even with limited resources, making them ideal for mobile applications, real-time systems, and tasks with a large number of input channels.

### 3.5. Proposed LightweightUNet Model

**Figure 5:**
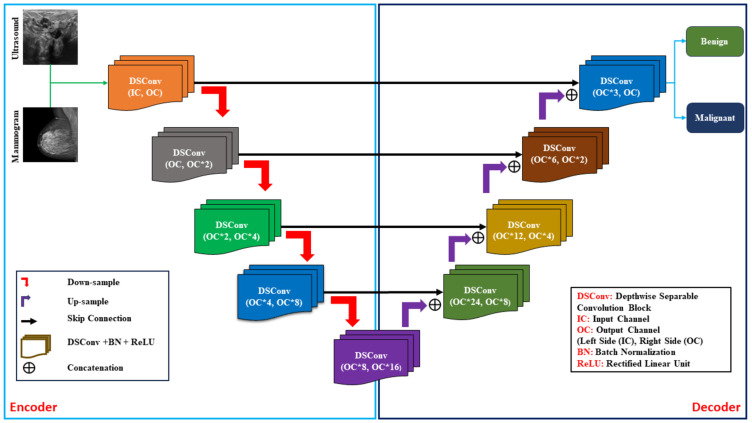
Architectural diagrams of the proposed LightweightUNet model for the detection of breast cancer.

Figure 5 illustrates our proposed LightweightUNet model used for detecting breast cancer using the multimodal (USI and MGI) dataset. We employed Depthwise Separable Convolutions (DSConv) blocks to design the model architecture. The proposed model consists of encoder and decoder sections, with DSConv blocks serving as the fundamental building blocks. To extract the fine details from the datasets, we employed a 3 × 3 kernel size in Depthwise convolution and a 1 × 1 kernel size in pointwise convolution, integral elements of the DSConv block. The architecture consists of only nine layers in its design, with four layers in the encoder section, four layers in the decoder section, and one layer in the bottleneck section. Due to its simplified structure and utilization of the DSConv block, the proposed model is adaptive, fast, has less computational cost, and can be deployed on portable devices.

#### 3.5.1. Encoder Section

The DSConv block, the core building component of the encoder, is found in the encoder section, where each layer adheres to a predetermined pattern. Two distinct convolutions are executed sequentially in this block. In the first, a 3 × 3 kernel size with stride of one and padding of 1 is used to analyze each channel of the input feature map individually in a Depthwise convolution. This essentially performs a per-channel filtering operation, extracting valuable spatial information within each channel. Mathematically, the Depthwise convolution operation can be represented by Equation (19).

(19) $$X_{d}\left(i,j,c\right)=\sum_{m=0}^{2}\sum_{n=0}^{2}X\left(i+m,j+n,c\right)\cdot K_{d}\left(m,n,c\right)$$

where Depthwise kernel value at position (*m, n*) in channel c is represented by $K_{d}\left(m,n,c\right)$ and X(i+m,j+n,c) is input feature map at $\left(i+m,j+n\right)$ in channel *c* (offset by kernel position). Following this, a pointwise convolution is applied. Unlike the Depthwise convolution, the pointwise convolution uses a 1 × 1 kernel to increase the number of output channels, effectively expanding the feature space to capture more complex information. In Equation (20), the pointwise convolution operation is represented and utilized in our process.

(20) $$X_{pw}\left(i,j,c_{out}\right)=\sum_{c_{in}=0}^{C_{in}-1}X_{d}\left(i,j,c_{in}\right)\cdot K_{p}\left(0,0,c_{in},c_{out}\right)$$

where the pointwise convolution kernel is $K_{p},$ the input feature map after depthwise convolution is $X_{d}$, and the output feature map is $X_{pw}.$ In our model architecture, Batch Normalization (BN) is a vital technique employed after each convolution within the DSConv block to ensure the stability of the training process. This method plays a crucial role in normalizing the activations across channels for each data point within a mini-batch. Mathematically, Batch Normalization involves the normalization, scaling, and shifting of the input feature map $Y_{pw},$ as expressed by Equations (21) and (22) [63,64].

(21) $${\hat{Y}}_{pw}=\frac{Y_{pw}-\mu_{B}}{\sqrt{\sigma_{B}^{2}+\epsilon }}$$

(22) $$Y_{BN}=\gamma {\hat{Y}}_{pw}+\beta$$

where $Y_{pw}$: input feature map, $\mu_{B}$: mini-batch mean, $\sigma_{B^{2}}$: mini-batch variance, and ϵ: small constant for stability; BN uses scale $\left(\gamma \right)$ and shift $\left(\beta \right)$ factors to normalize activation ${\hat{Y}}_{pw}$ by adjusting the mean and standard deviation. Following BN, a ReLU (Rectified Linear Unit) activation function is employed. This introduces non-linearity into the network, allowing it to learn more intricate relationships between features. The ReLU activation simply sets all negative values to zero while keeping positive values unchanged, introducing a threshold for feature selection (Equation (23)).

(23) $$Y_{relu}=\mathrm{ReLU}\left(Y_{bn}\right)$$

After every DSConv block, downsampling is used to process the relevant extracted features further. This technique reduces the feature maps’ spatial dimensions by a factor of 2. This enables the network to analyze larger image regions, allowing it to concentrate on gathering more complex semantic information. Downsampling is achieved by utilizing stride 2 and average pooling with 2 × 2 kernels. It can be expressed mathematically as given in Equation (24).

(24) $$Y_{\mathrm{down}}\left[i,j\right]=\frac{1}{4}\sum_{m=0}^{1}\sum_{n=0}^{1}Y_{\mathrm{conv}}\left[2i+m,2j+n\right]$$

where $Y_{\mathrm{down}}\left[i,j\right]$ denotes the downsampling feature map. The average pooling operation is applied along with the downsampling as given in Equation (25).

(25) $$D\left(Y\right)\left[i,j\right]=\frac{1}{k\cdot k}\sum_{m=1}^{k}\sum_{n=1}^{k}Y\left[i\cdot k+m,j\cdot k+n\right]$$

where $D\left(Y\right)\left[i,j\right]$ denotes the average pooling output. While downsampling reduces image resolution, it allows the network to gather deeper semantic features. This is beneficial for image classification and segmentation tasks. The encoder section gradually adds more layers to the output channels at each level. In order to produce a feature map with twice as many channels as the input picture, encoder 1 first takes the image with a specified number of channels. The output of the next three encoder levels (Encoders 2, 3, and 4) keeps on increasing by two times the number of channels above that of the preceding layer. As a result, the network is able to extract more intricate information from the input image progressively. The image resolution is decreased by downsampling using average pooling after each encoder block. This prepares the network to focus on capturing broader features in the subsequent decoder section. The combination of DSConv layers, BN, ReLU activations, and downsampling steps within the encoder section works together to efficiently extract informative features from the input image, laying the groundwork for accurate image analysis or segmentation tasks.

#### 3.5.2. Bottleneck (Center)

Within the LightweightUNet architecture, the bottleneck block, also known as the center block, bridges the encoder and decoder sections. It receives the output feature map from the final encoder block, encapsulating high-level semantic information. The bottleneck employs a Depthwise Separable Convolution (DSConv) Block, maintaining the same kernel size (3 × 3), stride (1), and padding (1) as used in the encoder. The bottleneck block significantly expands the feature representation by a factor of 2, resulting in 16 times the number of channels compared to its input (out_channels×8) to (out_channels×16), capturing a richer set of features before transitioning to the decoder for detailed image reconstruction. Mathematically, the bottleneck section can be represented by Equation (26) in this process.

(26) $$Y_{\mathrm{center}}=\mathrm{DSConv}\left(Y_{\mathrm{enc}4}\right)$$

Here, $\left(Y_{\mathrm{center}}\right)$ represents the output feature map of the bottleneck section. $\left(Y_{\mathrm{enc}4}\right)$ is the input from Encoder 4 with $\left(out_{c}hannels\times 8\right)$ channels. The Depthwise convolution operates with a 3 × 3 kernel, stride of 1, and padding of 1, while the pointwise convolution uses a 1 × 1 kernel to expand the number of channels. Batch normalization and ReLU activation are subsequently applied to ensure stability and introduce non-linearity. In essence, the bottleneck block acts as a vital bridge, efficiently processing high-level information. Crucially, it expands the feature space, preparing it for the decoder’s reconstruction tasks. This expansion facilitates a more robust and informative process in the subsequent decoder stages.

#### 3.5.3. Decoder Section

The LightweightUnet decoder section focuses on rebuilding the image’s spatial resolution and the level of detail visible. Unlike the encoder that extracted high-level features, the decoder progressively upsamples the feature maps, increasing their size. This upsampling is crucial, but it lacks the detailed information lost during downsampling in the encoder. To recover this lost detail, the decoder employs a clever technique called skip connections. In each layer, the upsampled feature map is “stitched” together with its corresponding feature map from the encoder. This combined feature map, with double the number of channels, now holds both the high-level information and the lost spatial details. Each decoder layer utilizes DSConv blocks, similar to the encoder. However, these DSConv blocks adjust their input channel configuration based on the number of channels resulting from the concatenation. Bilinear upsampling, a method for increasing image resolution, is performed before each DSConv block to enhance the spatial detail further. The upsampling operation utilized in our approach is presented by Equation (27).

(27) $${\hat{Y}}_{\mathrm{up}}\left(i,j\right)=\frac{1}{4}\left[Y\left(i,j\right)+Y\left(i+1,j\right)+Y\left(i,j+1\right)+Y\left(i+1,j+1\right)\right]$$

where $Y_{\mathrm{up}}$ is the upsampled feature map and $Y_{\mathrm{prev}}$ is the feature map from the previous decoder block. After upsampling, the concatenation operation is performed as given in Equation (28) in the decoder section after each layer.

(28) $$Y_{\mathrm{concat}}=\mathrm{Concat}\left(Y_{\mathrm{up}},Y_{\mathrm{enc}}\right)$$

where $Y_{\mathrm{concat}}$ is the concatenated feature map, $Y_{\mathrm{up}}$ is the upsampled feature map, and $Y_{\mathrm{enc}}$ is the corresponding encoder feature map. This combined feature map is then processed by a DSConv block, which can be mathematically expressed by Equation (29).

(29) $$Y_{\mathrm{dsconv}}=\mathrm{DSConv}\left(Y_{\mathrm{concat}}\right)$$

where $Y_{\mathrm{dsconv}}$ is the output feature map after applying the DSConv block; the decoder consists of four progressively shallower layers. Each layer starts by combining the output from the previous decoder block with its corresponding encoder feature map. This combined feature map is then processed by a DSConv block, which reduces the number of channels for the next layer. Through this combination of upsampling, skip connections, and DSConv blocks, the decoder meticulously reconstructs the image, incorporating both the high-resolution features from the encoder and the low-level details recovered during the upsampling process.

#### 3.5.4. Final Stage: Classification and Segmentation with Global Average Pooling

The final stage of LightweightUnet transforms the informative feature map from the decoder into a practical classification and segmentation mask. This mask precisely delineates objects of interest within the image. This crucial step involves two key components. The decoded feature map is first compressed using global average pooling. For each decoded feature map, this procedure averages across all spatial regions, reducing the spatial dimensions to a single value. It significantly reduces the computational cost by decreasing the number of elements the model needs to process. More importantly, it compels the model to focus on the most prominent features, discarding less relevant details. This allows the model to concentrate on the essence of the objects it aims to identify. The global pooling operation utilized for our approach is expressed by Equation (30).

(30) $$Y_{\mathrm{pooled}}=\frac{1}{H\times W}\sum_{i=1}^{H}\sum_{j=1}^{W}Y_{\mathrm{dec}}\left[i,j\right]$$

Here, $Y_{\mathrm{pooled}}$ represents the pooled feature map, $Y_{\mathrm{dec}}\left[i,j\right]$ provides feature map values at $\left(i,j\right)$ position, H represents the height, and W represents the width of the feature map. The fully connected (FC) layer receives the compressed feature vector $Y_{\mathrm{pooled}}$. The pooled feature vector is then handed over through an FC layer, which maps it to the number of output classes. For example, in binary segmentation tasks, where the output classes are binary (foreground and background), the FC layer maps the pooled feature vector to a single output neuron. The FC layer operation is expressed by Equation (31).

(31) $$\mathrm{Output}=W_{\mathrm{fc}}\cdot Y_{\mathrm{pooled}}+b_{\mathrm{fc}}$$

Here, $W_{\mathrm{fc}}$ represents the weight matrix of the FC layer, $b_{\mathrm{fc}}$ is the bias vector, and (Output) denotes the final segmentation output.

### 3.6. Assessment Metrics

We have used the standard assessment metrics listed below to validate the proposed method (Equations (32)–(37)).

(32) $$Accuracy\;(Acc)=\frac{TP+TN}{TP+TN+FP+FN}$$

(33) $$Recall\;(Rec)=\frac{TP}{TP+FN}$$

(34) $$Precision\;(Pre)=\frac{TP}{TP+FP}$$

(35) $$F1-score(F1)=\frac{2*Precision*Recall}{Precision+Recall}$$

(36) $$Weighted\;average=\sum_{i=1}^{N}\left(\frac{n_{i}}{N}\cdot {\mathrm{Metric}}_{i}\right)$$

(37) $$Macro\;average=\frac{1}{C}\sum_{i=1}^{n}\left({\mathrm{Metric}}_{i}\right)$$

where N represents the overall samples of the event and $n_{i}$ represents the cases in each class $i.\;{\mathrm{Metric}}_{i}$ denotes the calculated assessment metrics, precision, F1-score, and recall in each class i. The confusion matrix is the foundation for evaluating the classification task performed by the ML/DL models. In this matrix, the number of accurate classifications (*TP*, *TN*) and misclassifications (*FP*, *FN*) for an event is carefully presented. Where *TP*, *TN*, *FP*, and *FN* are true positive, true negative, false positive, and false negative, respectively. The confusion matrix takes on substantial significance when the effects of missing positive instances are considerable [65]. The presence of a high degree of recall value suggests that the model can lower the probability of missing important instances, even though it achieves it on account of a higher rate of false positives. It is crucial to maintain this balance since the risk of a false positive detection is smaller than that of a false negative [66,67].

## 4. Result

This section describes the experimental arrangement and obtained outcomes using the proposed methodology for detecting breast cancer. We have performed two separate experiments and used two different datasets: (1) the detection of breast cancer using the proposed model on the real dataset without augmentation, and (2) the breast cancer detection utilizing the combined dataset (real + GAN) using the proposed model. The first experiment utilizes 8000 mammogram images and 2400 ultrasound images, resulting in 10,400 images, for training the LightweightUNet model. The second experiment utilizes 8000 mammogram images and 12,400 ultrasound images (2400 original + 10,000 synthetic), resulting in 20,400 images. To validate the model performance, 2000 mammogram images and 600 ultrasound images are reserved as test datasets for both experiments.

The hardware components used for performing the experiments include a computer system with 16 GB random access memory (RAM), 1 TB Solid State Drive (SSD) storage capacity, and a 12 GB NVIDIA dedicated graphical processing unit (GPU). Given the large dataset consisting of 20,400 images with a resolution of 256 × 256, the SSD and GPU are essential for smoothly handling the data and accelerating the training process of the proposed deep learning model. The experiments were conducted in a Python programming environment utilizing the PyTorch framework. We optimized the various hyperparameters during the rigorous training process through a trial-and-error process and ultimately finalized the best hyperparameters for both experiments. A table summarizing the key hyperparameters used for both experiments, outlining differences in the input size, training type, kernel size, number of classes, learning rate, optimizer, batch size, and the number of epochs is presented in Table 3. These hyperparameters were meticulously calibrated to ensure robust model training and accurate breast cancer detection across diverse datasets and experimental conditions. The table highlights both similarities and differences in the configuration for each experiment, facilitating easier comparison and analysis of the results. Table 4 provides a comprehensive overview of the datasets employed for training and testing the LightweightUNet model. It details the distribution of images across both modalities (USI and MGI) and datasets (Real and combined (real + GAN) for the training and testing sets.

### 4.1. Breast Cancer Detection on Real Dataset (Without Augmentation)

The first experiment is performed on the real dataset without using data augmentation techniques utilizing the proposed LightweightUNet model. A multimodal approach utilizing two modalities (MGI and USI) was used for training and testing the proposed model. The MGI modality contains 10,000 images from three datasets, and the USI modality contains 3000 images from four datasets. Two pathology types (benign and malignant) are equally distributed in both datasets, with 5000 each in the MGI dataset and 1500 each in the USI dataset. The real dataset contains 13,000 images from both modalities, which were preprocessed and split into training and testing datasets. To avoid any potential bias, we have utilized an 80:20 train-test split of the real dataset. The training set contains 10,400 images (80% of the 13,000) from both modalities: 8000 images from the MGI modality, and 2400 images from the USI modality. The remaining 20% (2000 MGI images and 600 USI images) formed the unseen test set, totaling 2600 images. This unseen test set serves as a crucial evaluation tool, allowing us to assess the model’s generalization capability on data it has not encountered during training. The training data itself maintained a balanced class distribution, with 5200 benign images and 5200 malignant images. It is worth noting that due to the larger size of the MGI dataset, this modality dominates the training data. While this might influence the model’s initial learning towards MGI features, the chosen split and balanced training strategy aim to minimize this potential bias. The results of the 5-fold cross-validation training process are presented in the form of accuracy and loss curves. These visualizations provide valuable insights into the model’s learning behavior and its ability to improve its performance over training epochs.

**Figure 6:**
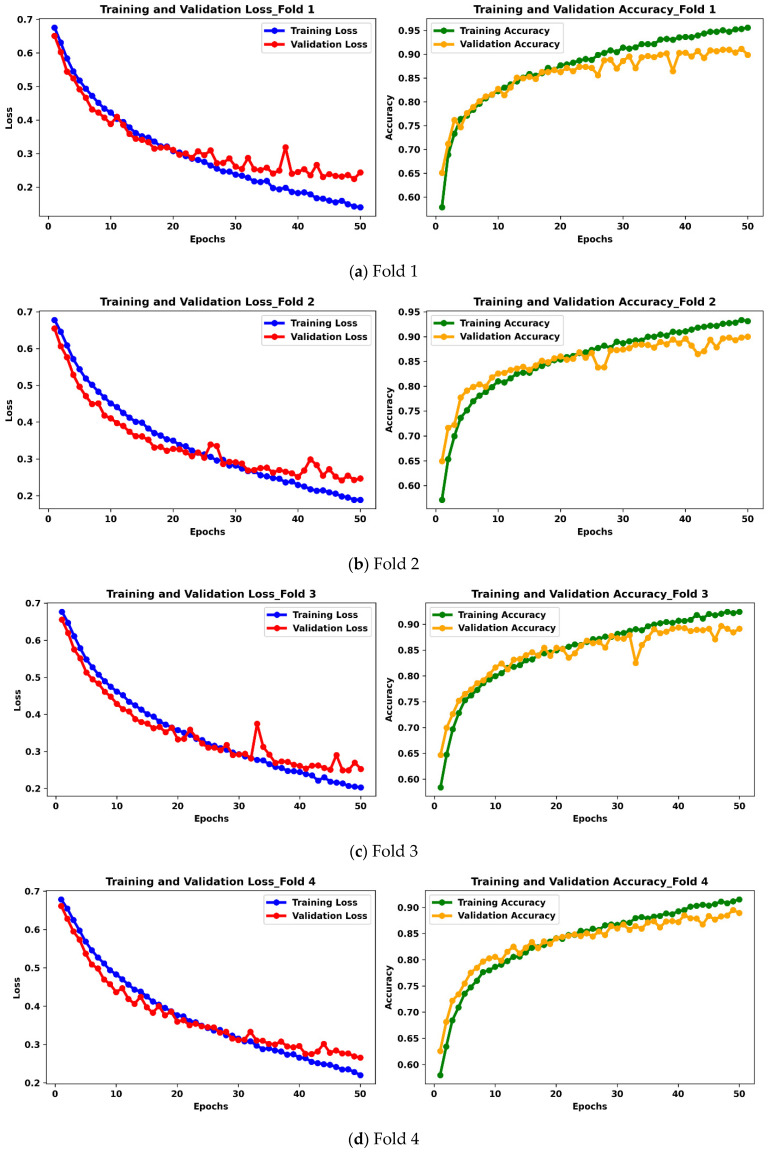

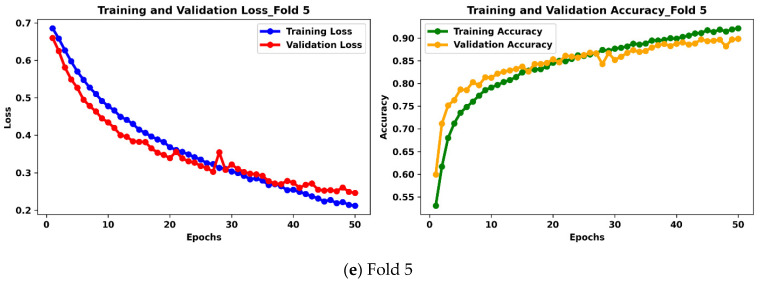
LightweightUNet training history on the real dataset (loss and accuracy).

Figure 6 visualizes the LightweightUNet classifier’s training procedure across all five folds of the 5-fold cross-validation on the real dataset. Each subplot (a–e) depicts a specific fold, showing the training (blue lines) and validation (red lines) curves for both loss and accuracy. Green lines denote training accuracy, while orange lines denote validation accuracy. All folds underwent training for 50 epochs.

- Early Folds (1 and 2): Subplots (a) and (b) reveal similar patterns for Folds 1 and 2. While training performance is promising (low loss and high accuracy), the validation curves exhibit wider gaps compared to later folds. For example, Fold 1 achieves a training accuracy of around 95%, but the validation accuracy shows a larger gap at the end of training. This could be a sign of overfitting, a condition in which a model performs well on unseen training data but poorly on validated data. The gap narrows slightly in Fold 2, indicating a possible improvement in generalization as training progresses.
- Later Folds (3–5): Subplots (c)–(e) depict Folds 3 to 5. These folds demonstrate a trend for better generalization. The gaps between validation and training curves for both loss and accuracy progressively narrow. Training accuracy stabilizes around 90% in these folds, with Fold 5 exhibiting the smallest gap and smoother curves. This shows that Fold 5 provides the best possible balance between applying what is learned from the training set to new data and retaining existing information. Furthermore, compared to previous folds, Folds 4 and 5 have smoother training and validation curves, suggesting a more robust learning process.

By examining these curves at every fold, we may learn a lot of information about how the model learns. During testing, we may observe how well it converges and how well it can generalize to new data. The model’s optimal weights from each fold, which indicate its state at maximum performance, have been saved for later analysis on test data that has not been seen. This allows us to assess the proposed model’s generalizability and overall detection accuracy over the entire real dataset.

**Figure 7:**
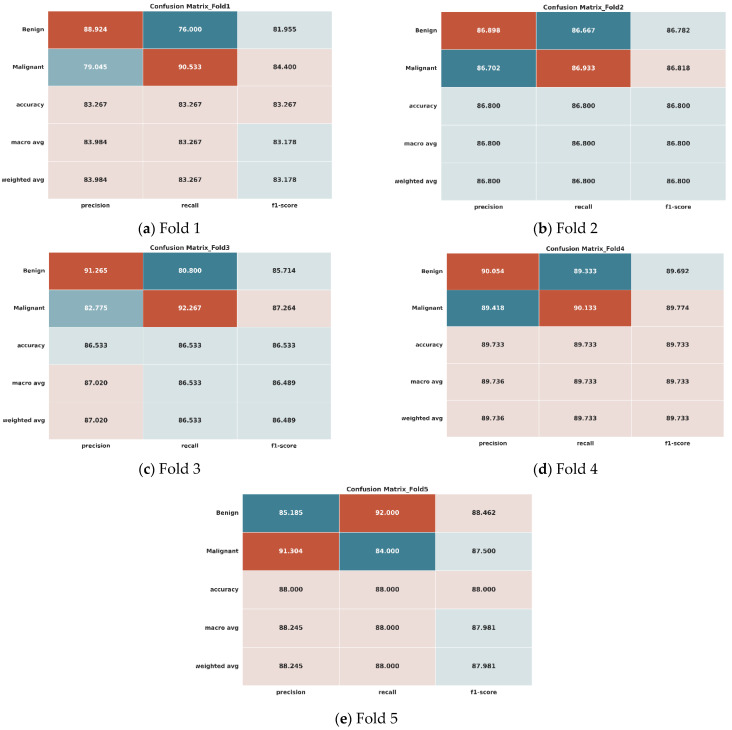
Breast cancer classification results using LightweightUNet (5-Fold CV) in terms of confusion matrix and metrics on a real dataset.

Figure 7 dives into the performance of the LightweightUNet model for breast cancer classification on a real dataset. Evaluated using 5-fold cross-validation, each subplot (a)–(e) represents a specific fold. These subplots showcase the classification results in two ways: a confusion matrix visualizing the distribution of true labels (benign or malignant) compared to the model’s predictions and various evaluation metrics like precision, F1-score, accuracy, recall, macro-accuracy, and average accuracy for both benign and malignant classes across all folds. Notably, the macro-average and weighted average values are identical due to the balanced nature of the testing dataset, containing an equal number of images (1300 each) from both benign and malignant classes, with a total size of 2600 images.

We have provided the classification outcomes of the initial experiment conducted on the real dataset, devoid of any augmentation, in Table 5. This table encapsulates the classification performances of both benign and malignant classes across each fold, delineating precision (Pre), recall (Rec), F1 score (F1), and accuracy (Acc). Additionally, we have computed the mean values of these metrics for both benign and malignant categories. Below, we delve into a detailed analysis of the table, discussing the observations for each fold individually and providing an overview of the collective results.

In the first fold, the model achieved a precision of 88.924% for benign cases, indicating a high level of accuracy in correctly identifying benign instances. The recall for benign cases was 76%, showing a moderate sensitivity in recognizing benign cases. The F1 score, which balances precision and recall, was 81.955%, reflecting a well-rounded performance. The overall accuracy for this fold was 83.267%. For malignant cases, the model had a precision of 79.045% and a high recall of 90.533%, resulting in an F1 score of 84.4%. The average metrics for this fold show balanced performance with an overall precision of 83.9845%, recall of 83.2665%, and F1 score of 83.1775%. Fold 1 demonstrated promising results, particularly in identifying malignant cases with high recall. However, there is room for improvement in the precision and F1 score for benign cases. In the second fold, the model’s performance was consistently high across both benign and malignant cases. For benign cases, the precision was 86.898%, with a recall of 86.667% and an F1 score of 86.782%, reflecting high accuracy and sensitivity. The overall accuracy was 86.8%. Similarly, for malignant cases, the precision was 86.702%, the recall was 86.933%, and the F1 score was 86.818%. The average metrics for this fold were 86.8%, showing consistent and reliable performance. Fold two exhibited consistent and reliable performance, with balanced metrics across both benign and malignant cases. The third fold showed very high precision for benign cases at 91.265%, though the recall was slightly lower at 80.8%, leading to an F1 score of 85.714%. The overall accuracy was 86.533%. For malignant cases, the precision was 82.775%, and the recall was very high at 92.267%, resulting in an F1 score of 87.264%. The average metrics for this fold were precision at 87.02%, recall at 86.5335%, and F1 score at 86.489%, indicating balanced and robust performance. Fold 3 demonstrated high precision for benign cases but showed slightly lower recall. However, it exhibited excellent sensitivity in detecting malignant cases. In the fourth fold, the model achieved a precision of 90.054% for benign cases, with a recall of 89.333% and an F1 score of 89.692%, reflecting high accuracy and sensitivity. In this fold, 89.733% overall accuracy was achieved. In malignant instances, the accuracy was 89.418%, the recall was 90.133%, and the F1 score was 89.774%. This fold has an average accuracy of 89.736%, a recall of 89.733%, and an F1 score of 89.733%, showing a stable and high-performing model. Fold four performed consistently well across all measures, demonstrating the model’s capacity to categorize both benign and malignant instances accurately. The fifth fold achieved high precision (85.185%) for benign cases, along with a very high recall (92%), resulting in a strong F1 score (88.462%). In this fold, 88% overall accuracy was achieved. For malignant instances, the model maintained high accuracy (91.304%) but somewhat reduced recall (84.0%), resulting in an F1 score of 87.5%. The model performed well in classification, with an average accuracy of 88.2445%, a recall of 88.0%, and an F1 score of 87.981%. Notably, Fold Five performed exceptionally well in recognizing benign cases with good recall while also attaining balanced performance in malignant case categorization.

Across all folds, the proposed model showed a good overall performance. The overall performance is calculated by averaging the values of metrics from each fold for each pathology, benign and malignant. For the benign class, the average precision, recall, and F1 score were 88.4652%, 84.96%, and 86.521%, respectively. For malignant cases, the average precision of 85.8488%, recall of 88.7732%, and F1 score of 87.1512% were observed. The model’s robustness and dependability in classifying breast cancer into benign and malignant categories using the real dataset have been verified by the overall average precision (87.157%), recall (86.8666%), F1 score (86.8361%), and accuracy (86.867%). It is observed that among all folds, fold three performed exceptionally well in correctly classifying instances of breast cancer and produced the highest accuracy of 89.73%. Meanwhile, the proposed LightweightUNet model produces an overall accuracy of 86.867%. All folds consistently obtain good accuracy, precision, recall, and F1 scores for the proposed LightweightUNet model, as shown by the experimental result of each fold and the overall performance metrics. The LightweightUNet model is an efficient model for classifying breast cancer, and it showed consistently good performance across all folds. Recall of 86.8666%, accuracy of 86.867%, precision of 87.157%, and F1 score of 86.8361% indicate an excellent performance by the proposed approach overall. But there is still much space for growth, indicating directions for more development and improvement.

### 4.2. Breast Cancer Detection on Real + GAN Images

The second experiment delves deeper into the capabilities of the proposed LightweightUNet model by evaluating its performance on a dataset augmented with synthetic ultrasound (USI) images. Since the existing real USI dataset, denoted as MGI, contained a sufficient amount of data (8000 images) for model training, we opted to leverage the power of StyleGAN3, a generative adversarial network, to create an additional 10,000 high-quality synthetic USI images. This data augmentation strategy aimed to enrich the training dataset and potentially improve the model’s ability to learn complex patterns from USI data. The inclusion of these synthetic images significantly increased the total USI dataset size to 12,400 images (2400 real+ 10,000 synthetic). When combined with the original 8000 mammogram images (MGI), the entire training dataset for this experiment comprised 20,400 images. Notably, we maintained a balanced class distribution within the training set, ensuring there were 10,200 images each for benign and malignant cases. This balanced distribution is crucial for training machine learning models to avoid biases towards one class over another. It is important to emphasize that the test dataset (2600 images containing both modalities) remained unchanged from the first experiment. We deliberately refrained from introducing any synthetic data into the test set to ensure consistency with the initial evaluation process. This allows for a direct comparison of the model’s performance on unseen data (real test set) across both experiments, one with data augmentation and one without. While the class distribution within the training set is balanced, there exists a slight imbalance in the overall data size between the two modalities (MGI: 8000 vs. USI: 12,400). However, this difference allows us to analyze the impact of a larger training dataset on the model’s performance compared to the first experiment, where data augmentation was not employed. The results of the 5-fold cross-validation training process, including visualizations of accuracy and loss curves, will be presented in a later section. These visualizations offer valuable insights into the model’s learning behavior, particularly its ability to learn and improve its performance over the 50 training epochs.

**Figure 8:**
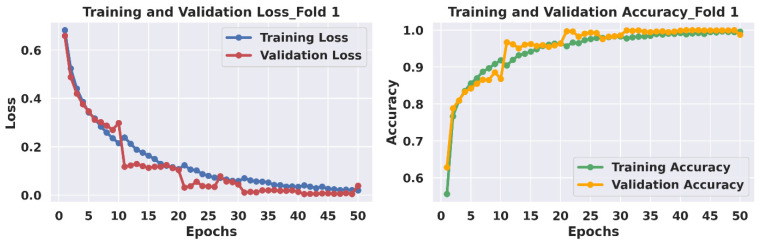

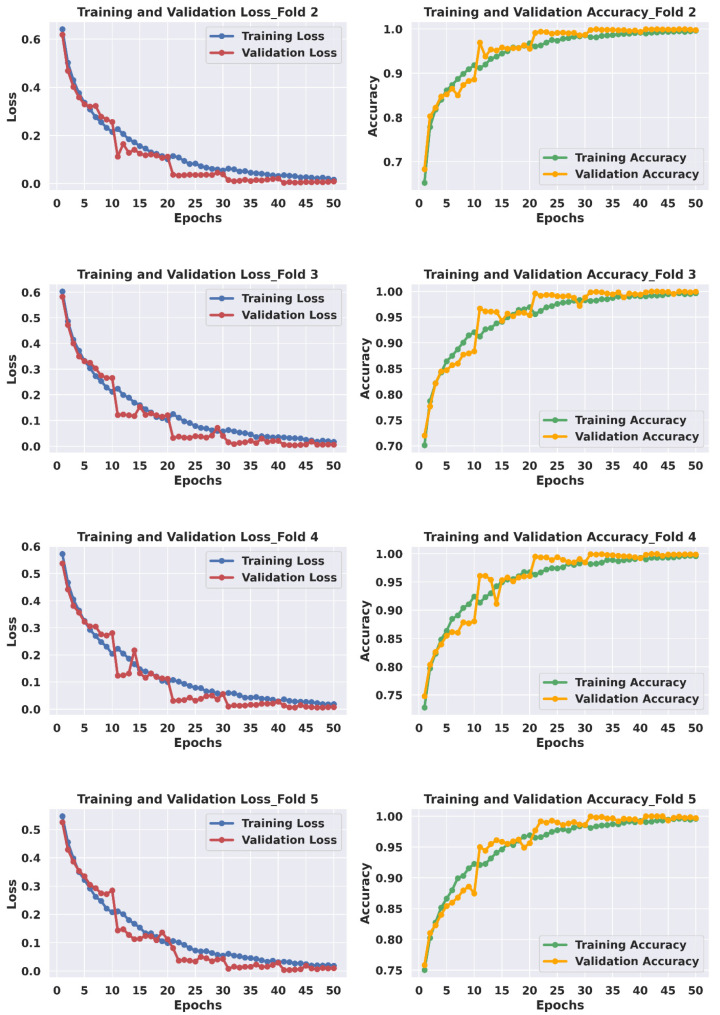
LightweightUNet training history on real + GAN dataset (loss and accuracy).

We have illustrated the training history of each fold, capturing loss and accuracy on both validation and training datasets using our LightweightUNet model in Figure 8. Analyzing these curves reveals several key observations:

- Convergence: The loss curves for all folds exhibit a clear downward trend, approaching near-zero values by the end of the training process (50 epochs). This indicates successful model training, as the model minimizes the loss function and learns to classify breast cancer cases accurately.
- High Accuracy: As training progresses, the accuracy curves for the validation and training datasets both show substantial improvement, potentially approaching 100%. This suggests that the model achieves a high degree of accuracy in classifying breast cancer on both the training data and validation data used for evaluation.
- Validation Fluctuations: While the accuracy curves for training data show a smoother progression, the validation curves exhibit some initial fluctuations. However, these fluctuations stabilize towards the end of training, indicating that the model’s performance on unseen data becomes more consistent.
- Closing Performance Gap: It is noteworthy that the gap between training and validation performance narrows significantly throughout training. This suggests that the model is generalizing well and avoiding overfitting the training data. In some cases, the validation accuracy even surpasses the training accuracy during the training process.
- Impact of GAN-Generated Data: These observations can be attributed, in part, to the addition of GAN-generated high-quality synthetic data in the training set. The extra data likely provided two key benefits: Increased Data Volume and Enhanced Generalizability. Firstly, the additional images augmented the overall dataset size, affording the model a broader spectrum of training examples to learn from. Secondly, the synthetic images, meticulously crafted to emulate real USI data, introduced variations and intricacies that enriched the learning process. Consequently, the model became better equipped to generalize its learning to unseen data, as evidenced by its performance on the validation set.

Overall, the training history suggests successful model training with good convergence and high accuracy. The inclusion of GAN-generated data has contributed to improved model performance and generalizability.

**Figure 9:**
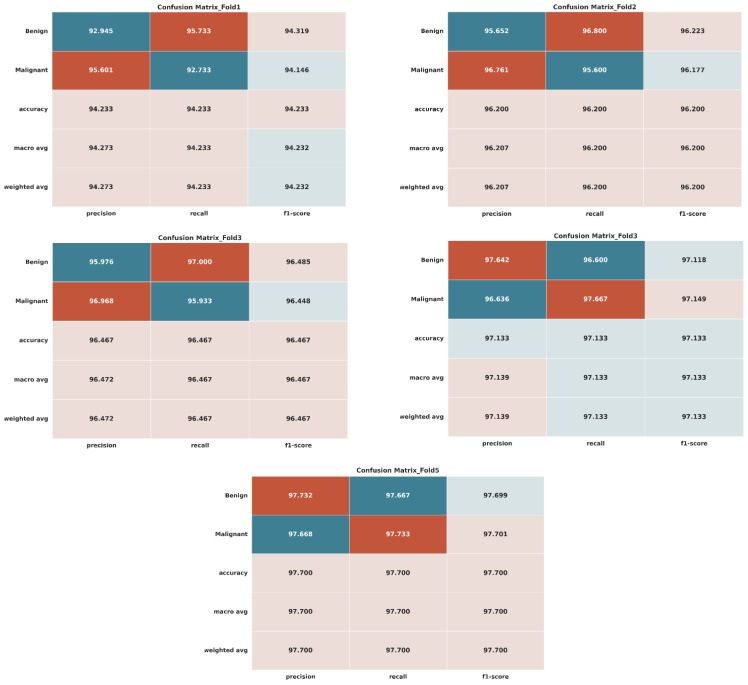
Breast cancer classification results using LightweightUNet (5-Fold CV) in terms of confusion matrix and metrics on real + GAN dataset.

Figure 9 offers a visual exploration of the LightweightUNet model’s performance on the real + GAN dataset for breast cancer classification, evaluated using 5-fold cross-validation. Each subplot (a)–(e) corresponds to a specific fold and presents the classification results through two key visualizations: confusion matrix and evaluation metrics. The confusion matrix provides a clear visual representation of how well the model’s predictions (benign or malignant) align with the actual labels present in the test data for that specific fold. Analyzing these matrices across folds can reveal potential biases or class imbalances in the model’s predictions.

Table 6 provides a more detailed quantitative assessment of the performance of the LightweightUNet model using the real + GAN dataset. The performance metrics (precision, recall, accuracy, and F1-score) for the benign and malignant classes are shown in the table for each of the five folds that were utilized during the cross-validation procedure. This decomposition enables an improved understanding of the model’s performance in distinguishing between benign and malignant classes within each fold. In addition, we computed the average values of these measures for the categories of benign and malignant cases. Comparing the model’s performance with other studies or other models is made easier by analyzing these mean values, which also give a general view of the model’s performance over the entire test set.

By analyzing the result in Table 6, we can observe that, in the initial fold, the model showcased remarkable performance across all metrics. The accuracy of the classification of both forms of cancer has been demonstrated with 92.945% precision for benign cases and 95.601% precision for malignant instances. With benign case recall rates of 95.733% and malignant case recall rates of 92.733%, the recall rates also appeared stronger. With 94.319% for benign instances and 94.146% for malignant cases, the F1 scores were consequently quite high. This fold has an overall accuracy of 94.233%. Setting a high bar with precision, recall, and F1 scores close to 95% for both benign and malignant patients, the first fold demonstrated the model’s exceptional performance. The mean values for precision, recall, F1 score, and accuracy were 94.273%, 94.233%, 94.2325%, and 94.233%, respectively, across all folds. The second fold continued the pattern of exhibiting very high performance levels. With benign cases reaching 95.652% and malignant cases reaching 96.761%, precision remained high. Recall rates were also strong, reaching 95.6% for malignant cases and 96.8% for benign instances. An overall accuracy of 96.2% was obtained from the remarkable F1 scores, which were 96.223% for benign patients and 96.177% for malignant cases. By demonstrating the model’s ability to consistently attain high accuracy, recall, and F1 scores across several dataset subsets, Fold 2 confirmed the model’s consistency and dependability. Through all folds, the average values for precision, recall, F1 score, and accuracy were 96.2065%, 96.2%, 96.2%, and 96.2%, respectively. The model continued to perform exceptionally well in the third fold, sustaining accuracy values of 96.968% for cases of tumors and 95.976% for benign instances. With 97% of cases being benign and 95.933% of malignant cases, recall rates were still high. Notable F1 scores were obtained, with benign patients scoring 96.485% and malignant ones scoring 96.448%. For this fold, the total accuracy was 96.467%. Fold 3′s performance was constant and dependable; its high recall rates were especially notable and indicated the model’s capacity to capture real positive cases accurately. The average values for accuracy, F1 score, precision, and recall were 96.4665%, 96.4665%, 96.4665%, and 96.4665%, respectively, for all folds. With precision levels of 96.636% for malignant instances and 97.642% for benign cases, the fourth fold carried on the remarkable performance trend. The recall rates were 96.6% and 97.667% for benign and malignant cases, respectively. The F1 scores obtained for both benign and malignant cases are 97.118% and 97.149%, respectively, and the overall accuracy is 97.133%. Fold 4 showed that the model can consistently obtain high recall and accuracy, indicating that it was effective in correctly identifying benign and malignant classes. The average score for accuracy, precision, recall, and F1 score were 97.139%, 97.1335%, 97.1335%, and 97.1335%, respectively, across all folds. In the 5th Fold, the model performed extremely well, with accuracy scores of 97.732% for benign cases and 97.668% for malignant cases. The recall values were 97.667% for benign cases and 97.733% for malignant cases. The F1 score in this fold is also high, with 97.699% for benign patients and 97.701% for malignant patients, with an overall accuracy of 97.7%. The average precision, recall, F1-score, and accuracy achieved 97.7% in this fold, which validates the model’s robustness and reliability through 5-fold cross-validation, demonstrating consistently high performance.

The LightweightUNet model’s average metrics overall folds, when used for the classification of breast cancer on a combined real and synthetic (GAN-generated) dataset, show encouraging outcomes. For both benign and malignant categories, the model performed excellently. In terms of Benign Classification, the model showed great accuracy (95.99%), which means that approximately 96% of all positive predictions for benign instances were accurate. It also demonstrated a high recall of 96.76%, meaning that the model correctly classified more than 96% of real benign instances. The F1-score of 96.37% shows a balanced performance between recall and accuracy. Lastly, the model’s total 96.35% accuracy rate indicates how well it can categorize benign samples. Similar favorable findings were seen for malignant patients. The model has a high accuracy of 96.73%, implying a high percentage of correct positive predictions. The recall of 95.93% implies that the model can detect more than 95% of malignant instances. While the F1-score of 96.32% is significantly lower than in benign situations, it still demonstrates excellent performance. Overall Performance (Benign and Malignant): The model’s average precision (96.36%) demonstrates its excellent accuracy in positive predictions across all instances. The outstanding average recall of 96.35% demonstrates its ability to detect both benign and dangerous tumors. Finally, the average F1-score of 96.35% demonstrates well-balanced performance across many criteria. Using the combined dataset, the LightweightUNet model performs remarkably well in the classification of breast cancer. All measures for benign and malignant instances had consistently high values, indicating the robustness and dependability of the model in differentiating between these categories. The balanced F1 scores reinforce the finding. LightweightUNet appears to be a very useful tool for classifying breast cancer based on these data, possibly helping with fast and precise diagnosis—a crucial step toward better patient outcomes.

### 4.3. The Effect of GAN Images on Breast Cancer Detection

**Figure 10:**
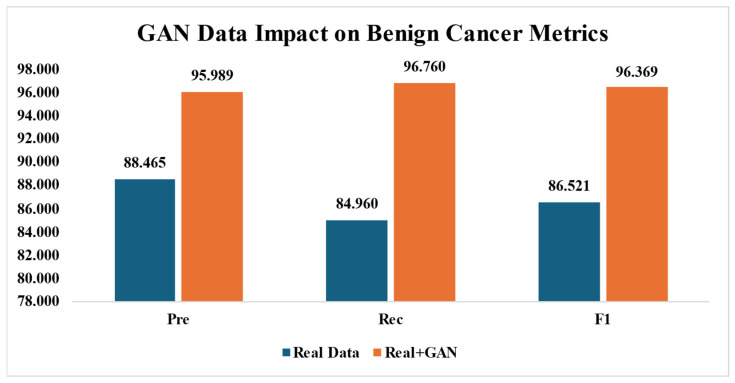
Impact of GAN-generated data on breast cancer classification for the benign class using LightweightUNet model.

We examined the effect of GAN data on breast cancer diagnosis after assessing and analyzing the outcomes of classification for both research projects utilizing real data and the combined (real + GAN) data. In particular, we evaluated the benign class’s classification performance in terms of F1 score, accuracy, and recall. The impact of GAN-generated data on the classification accuracy of benign breast cancer patients using the LightweightUNet model was assessed by an experiment. Precision, recall, and F1-score for classification results were compared across all folds for both experiments, one with real data only and the other with a combined dataset (real + GAN). Using GAN-generated data produced notable gains in all three benign class categorization criteria, as Figure 10 illustrates. With real + GAN data, the model’s accuracy rose from 88.465% to 95.989%, indicating a 7.52% improvement in its ability to identify true benign instances correctly. In addition, the recall of the model for benign patients significantly increased, rising from 84.960% with actual data to 96.760% with real + GAN data. This is an 11.80% increase, suggesting a higher capacity to identify real benign instances. An additional noteworthy improvement can be seen in the F1-score, which weighs recall and precision equally. It improved from 86.521% with actual data to 96.369% with real + GAN data. This 9.85% increase suggests a better balanced performance between recall and accuracy. The model’s capacity to classify instances of benign breast cancer was much improved by adding GAN-generated data, as seen by the overall considerable gains across all measures. The proposed model displayed increased recall values for benign classes, and it also provided a high-precision value, minimizing false positives while maintaining an overall balanced performance as measured by the F1-score.

**Figure 11:**
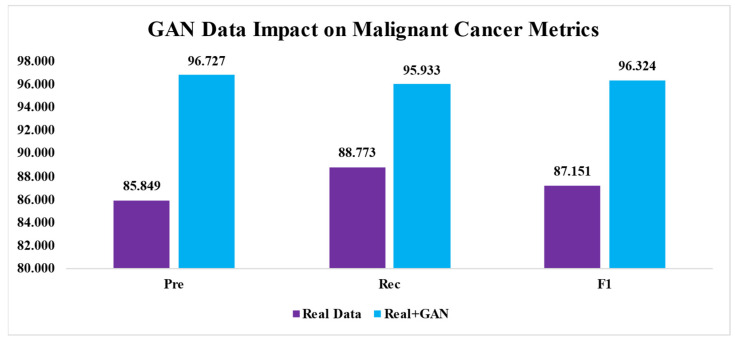
Impact of GAN-generated data on breast cancer classification for the malignant class using LightweightUNet model.

Figure 11 demonstrates the impact of synthetic data generated using styleGAN3 with respect to precision, recall, and F1 score metrics using the proposed LightweightUNet model for classifying the malignant classes. The model achieved an 85.849% precision value on the real dataset and 96.727% on the combined dataset, with an improvement of 10.88%. The recall value on the real dataset was 88.773% and improved to 95.933% on the combined datasets, showing a 7.16% improvement. Similarly, the F1 score also increased to 96.324% on a combined dataset from 87.151% on the real dataset, with an increase of 9.17%. These values show that the impact of synthetic data for detecting the malignant classes is high in terms of all metrics values.

**Figure 12:**
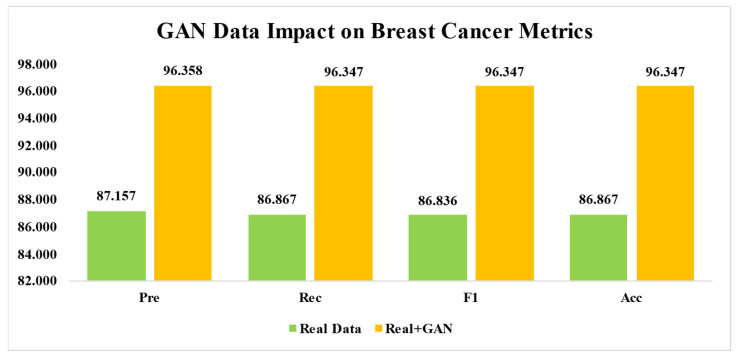
Impact of GAN-generated data on breast cancer classification metrics for breast cancer detection using LightweightUNet model.

Finally, we have analyzed the overall performance by combining pathology types (benign and malignant) and the impact of the GAN-generated data on the proposed LightweightUNet model. The overall performance of the model is compared in terms of all four metrics: precision (Pre), recall (Rec), F1-score (F1), and accuracy (Acc). Figure 12 presents the model’s performance on real data and combined data (real + GAN). According to the results obtained, GAN-generated data significantly improves the performance of the model in terms of all metrics detailed as follows:

- Precision: The model obtained 96.358% accuracy using actual + GAN data, against 87.157% with real data only. This corresponds to a 9.20% improvement, suggesting a greater capacity to reliably distinguish real breast cancer patients from all positive predictions.
- Recall: The model’s ability to identify actual cases of breast cancer also significantly improved. The recall improved by 9.48%, from 86.867% with actual data to 96.347% with real + GAN data.
- F1-Score: A comparable improvement may be seen in the F1-score, which strikes a balance between recall and accuracy. With actual + GAN data, it improved by 9.51%, from 86.836% with real data to 96.347%. This implies a more evenly distributed performance overall.
- Accuracy: Notably, there was a notable improvement in the model’s overall accuracy as well. Using actual + GAN data, the accuracy of the model was 96.347%, but using real data alone, it was only 86.867%. This corresponds to a 9.48% improvement, suggesting a stronger capacity to categorize instances accurately.

These large increases in all measures clearly imply that the model’s capacity to classify instances of both benign and malignant breast cancer was much improved by the incorporation of GAN-generated data. The model retained a balanced performance (F1-score) and became more accurate in recognizing real positives (precision) and a greater proportion of real instances (recall). The present study demonstrates the potential of GAN-generated data to supplement the LightweightUNet model’s training process, resulting in a more dependable and durable tool for breast cancer diagnosis.

### 4.4. Computational Complexity

Our proposed LightweightUnet model for breast cancer detection on a multimodal dataset consists of only nine layers, designed for efficient detection with low computational complexity. The architecture includes the DSConv in both the encoder and decoder sections, which reduces the computational liability as compared to the standard convolutional layer. Since we have used small kernel sizes in this model (3 × 3 for Depthwise convolution and 1 × 1 for pointwise convolution), the reduction in the computational load is substantial, especially as the input and output channels increase. The model includes only four layers in the encoder section, four layers in the decoder sections, and one bottleneck layer; it features downsampling and bilinear upsampling to process images, while batch normalization ensures stability efficiently. The LightweightUNet model architecture is in U-Net shape, which reduces computational complexity with its encoder and decoder architecture with skip connections. This design not only ensures efficient feature extraction and image reconstruction but also minimizes the number of required operations, which makes the model achieve better results along with a low computational cost.

We have also included the training time and testing time taken by the proposed model on the training and testing dataset to produce the result utilizing GPU and CPU processors, as presented in Table 7. However, a direct comparison of the computational complexity with the state-of-the-art model was not feasible due to the multimodal dataset and 5-fold cross-validation training, as it is not feasible to train the existing models on multimodal data without modifications.

For training, the computational complexity is influenced by the image size (256 × 256) and the model structure. Utilizing GPU, the model takes 91 min to train for 50 epochs across all five-fold cross-validations. This result reflects the high efficiency of the DSConv blocks in reducing the computational load, allowing faster processing with the GPU. On the CPU, the training time increases to 162 min, which indicates that the GPU provides a substantial speedup due to its parallel processing. The training time taken by the proposed model is much less than that of many traditional models in general. For the testing phase, computational costs are minimal, with GPU testing taking just 0.00392 min and CPU testing taking 0.00627 min. As the model is already trained, testing simply involves making predictions. In this testing phase, the trained model is tested on the unseen test dataset. As the LightweightUNet model has fewer layers and utilizes more efficient convolution operations, this enables fast inference times. Overall, the proposed LightweightUNet model demonstrates low computational complexity during training and testing, making it suitable for deployment on resource-constrained devices like portable and mobile devices.

## 5. Discussion

This section explores the performance of several methods for classifying breast cancer, as illustrated in Table 8. Including studies performed between 2020 and 2024, this table offers a useful overview of current developments in the subject. We have performed a comparative study based on a number of important factors, including the evaluation criteria applied, the techniques used, the datasets utilized in training, and the imaging modalities included.

A number of studies, such as those by Vaka et al. [68] with DNNS (97.21%), Kavitha et al. [69] with BPNN (98.50%), Ragab et al. [70] with CSO-MLP (97.09%), Asadi and Memon [26] with U-Net + ResNet50 (96.80%), Sheeba et al. [24] with TCL-RAM (97%), Haris et al. [71] with SVM (98.83%), Sahu et al. [28] with ResNet18, MobileNetV2, AlexNet (97.50%), and the proposed LightweightUNet (96.36%), achieved high accuracy in classifying breast cancer.

But it is imperative to go beyond accuracy alone. It is crucial to achieve an equilibrium between recall (identifying all malignant tissue) and precision (identifying malignant tissue properly) in real-world medical situations. A clear benefit may be shown in this area with the proposed LightweightUNet model. Its success in capturing true positives—that is, cases that are properly identified as malignant—and limiting false positives—that is, instances that are mistakenly categorized as benign—is indicated by its well-balanced performance with accuracy (96.36%), recall (96.35%), and F1-score (96.35%). The possibility of missed diagnoses, which can have serious consequences for patients, must be reduced with the help of this balanced approach.

**Table 8:** Performance evaluation of the proposed LightweightUNet model for breast cancer classification on the multimodal dataset.

Literature	Year	Model Used	Modality	Dataset Utilized	Metrics
[68]	2020	DNNS	H&E	Private dataset	0.9721 (Acc), 0.9790 (Pre) 0.9701 (Rec)
[17]	2020	VGG16 + SVM	H&E	BreakHis	0.9397 (Acc), 0.9400 (F1), 0.9400 (Pre), 0.9300 (Rec)
[18]	2021	FC-DSCNN	MGI	PINUM	0.9000 (Acc), 0.8500 (F1), 0.8900 (Pre), 0.8200 (Rec), 0.9900 (Sen), 0.8200 (Spe)
[69]	2022	BPNN	MGI	DDSM	0.9850 (Acc), 0.9891 (F1), 0.9846 (Sen), 0.9908 (Spe)
[70]	2022	CSO-MLP	USI	BUSI	0.9709 (Acc), 0.9476 (Pre), 0.9554 (Sen), 0.9765 (Spe)
[25]	2023	CBR + SE-ResNet101	MGI	CBIS-DDSM	0.8671 (Acc), 0.7500 (F1), 0.6400 (IoU), 0.8100 (Pre), 0.7600 (Rec)
[24]	2023	TCL-RAM	H&E	Break His	0.9700 (Acc), 0.9300 (Sen), 0.9400 (Spe)
[72]	2023	Ensemble Classifier	MGI	DDSM	0.9326 (Acc), 0.8989 (F1), 0.9040 (Pre), 0.8231 (Rec), 0.9320 (Spe)
[26]	2023	ResNet50	MGI	INbreast,	0.9680 (Acc), 0.9680 (F1), 0.9680 (Pre), 0.9680 (Rec)
[73]	2024	DualNet-DL model	MGI	CBIS-DDSM	0.9429 (Acc), 0.9579 (F1), 0.9832 (Pre), 0.9474 (Sen), 0.9474 (Spe)
[28]	2024	ResNet18, MobileNetV2, AlexNet,	MGI, USI	BUS2	0.9750 (Acc), 0.9749 (F1), 0.9798 (Pre), 0.9700 (Sen), 0.9800 (Spe)
[71]	2024	SVM	MGI	CBIS-DDSM	0.9883 (Acc), 0.9883 (F1), 0.9883 (Pre), 0.9883 (Rec)
[30]	2024	TwinCNN	H&E MGI	BreakHis	0.9770 (H&E Acc), 0.9130 (MGI Acc), 0.6840 (Fused Acc)
[32]	2022	MobileNet CNN (Lightweight DNN)	H&E	Private Dataset	0.8750 (Acc)
[33]	2023	Lightweight Separable Convolution (LWSC)	H&E	BreaKHis	0.9723(Acc), 0.9771 (Sen), 0.9793 (Spe)
[34]	2023	PLA Framework	H&E	BreakHis	0.953 (Acc), 0.969 (F1), 0.988 (Pre), 0.998 (Rec)
[35]	2023	Lightweight CNN	H&E	ICIAR 2028, BreakHis, Bracs	0.9625 (Acc) ICIAR 2018, 0.998 (Acc) BreakHis, 0.722 (Acc) Bracs
[38]	2024	Breast-NET + Transfer Learning (Lightweight DCNN)	H&E	BreakHis	0.9034 (Acc), 0.89 (F1), 0.86 (Pre), 0.92 (Rec)
[39]	2024	ResNet34 + SVM (Lightweight Technique)	Thermography Images	DMR-IR database	0.9962 (Acc), 0.9963 (F1), 0.9963 (Pre), 0.9963 (Rec)
[40]	2025	Multi-light Net (multi-input lightweight CNN)	Thermography Images	DMR-IR database	0.9814 (Acc), 0.9726 (F1), 1.0 (Pre), 0.9467 (Rec), 1.0 (Spec)
Proposed	2024	LightweightUNet	MGI, USI	BrEaST, BUSI, Thammasat, HMSS, DDSM, MIAS, INbreast	0.96358 (Acc), 0.96347 (F1), 0.96347 (Pre), 0.96347 (Rec)

Abbreviations: H&E—Hematoxylin and Eosin, DNNS—Deep Neural Network with Support Value, FC-DSCNN—fully connected depth-wise-separable convolutional neural network, BPNN—Back Propagation Neural Network, MGI—Mammography Imaging, DDSM—Digital Database for Screening Mammography, BUSI—Breast Ultrasound Image dataset, CSO—Cat Swarm Optimization, MLP—Multilayer Perceptron, CBIS-DDSM—Curated Breast Imaging Subset of DDSM, BCDR—Breast Cancer Digital Repository, MIAS—Mammographic Image Analysis Society, USI—Ultrasound Imaging, TCL-RAM—transfer learning integrated with regional attention mechanism, SVM—Support Vector Machine, PLA -Privacy-Embedded Lightweight and Efficient Automated.

A model’s generalizability, or its performance on unseen data, is greatly affected by the quantity and variety of training data. Most studies included in our comparison leveraged multiple datasets for breast cancer classification, with a few exceptions. For instance, Vaka et al. [68] utilized a private hospital dataset (Sharma and Mehra, 2020) [17] used the BreakHis open-access dataset (Ragab et al., 2022) [70] employed the BUSI public dataset, and both (Bouzar-benlabiod et al., 2023) [25] and (Haris et al., 2024) [71] used the CBIS-DDSM dataset. In contrast, our proposed technique stands out for its comprehensive use of datasets from six different sources: BrEaST, BUSI, Thammasat, HMSS, DDSM, MIAS, and INbreast. Furthermore, we included a substantial amount (10,000) of synthetic images created using GANs. This large and varied dataset approach improves our model’s robustness and practical applicability because it can better manage the differences in image acquisition methods that are frequently used in clinical situations.

Most of the studies employed mammography images (MGI) as the primary modality; a few of them also explored H&E and ultrasound (USI). As an illustration, Sahu et al. [28] employed many datasets with MGI and USI modalities. However, they carried out their studies independently for each dataset and modality. In a similar vein, Oyelade et al. [30] conducted studies independently on each of the two datasets—H&E and MGI—as well as on a combined (fused) modality. They discovered that the combined modality yielded worse results for H&E (68.40%) than for MGI (91.30%) and H&E alone (97.70%). The limitation of this study is that the multimodal approach does not show improved performance, which shows that their proposed model is not capable of learning the features from both modalities.

Lightweight models have made significant improvements in detecting breast cancer across a variety of datasets and modalities, as shown in several studies that we have utilized in the comparison table. The models were developed to boost accuracy and computing efficiency, which makes them suitable for practical applications. Nneji et al. [33] used the LWSC method for breast cancer categorization, and they achieved an accuracy of 0.9723 with high sensitivity and specificity (0.9771 and 0.9793, respectively), while Laxmisagar et al. [32] utilized MobileNet CNN on H&E images and achieved an accuracy of 0.8750. On the other hand, Yan et al. [34] employed the PLA framework to obtain an accuracy of 0.953 on the BreakHis dataset, and Kausar et al. [35] obtained great results on various datasets, including 0.998 on BreakHis and 0.9625 on ICIAR 2018, although they achieved an accuracy of 0.722 on Bracs. Saha et al. [38] applied transfer learning with Breast-NET and obtained an accuracy of 0.9034 on the BreakHis dataset, with precision and recall scores of 0.86 and 0.92, respectively. Nguyen Chi et al. [39] produced an impressive 0.9962 accuracy, 0.9963 precision, recall, and F1 score for thermography image classification using ResNet34 and SVM, whereas Elaraby et al. [37] used a Lightweight CNN (LWCNN) to attain an accuracy range of 0.9500 to 0.0999. In the end, Tang et al. [40] used Multi-light Net for lightweight CNNs with multiple inputs. They accomplished outstanding outcomes on the DMR-IR database, reaching an accuracy of 0.9814, precision of 1.0, and specificity of 1.0.

Unfortunately, none of these methods have a holistic approach, which is a big problem. Most studies fail to integrate various modalities to enhance detection accuracy, instead relying on single-modality data like H&E images, thermal images, or any other kind of imaging. Existing lightweight models for breast cancer detection have not fully investigated the possibility of improving model performance through the utilization of complementary information. One approach that could be considered is a multimodal approach, which involves combining various imaging techniques such as thermography, mammography, ultrasound, and histopathology.

In this work, we performed the experiments utilizing a multimodal approach (MGI and USI) using the proposed LightweightUNet model. We obtained an exceptional accuracy of 96.34% on a combined dataset for detecting breast cancer. This shows that our model is very good at extracting the features from multimodal data, especially in the case of breast lesion detection. Also, our model achieved overall precision (96.36%), recall (96.35%), and F1-score (96.35%) on the combined dataset using the proposed LightweightUNet model. Also, the combined dataset showed a minimum 9% improvement of all metrics because of the impact of the generated dataset.

The datasets used for this study, which were from seven different sources, exhibit inherent variability. At the same time, the data from the two imaging modalities, such as mammograms and ultrasounds, possess general characteristics; small variations in data preparation regarding acquisition protocols, resolution, and preprocessing by publishers might influence the performance of and generalizability of the model proposed, called LightweightUNet. These problems were resolved by resizing all images to a standard resolution of 256 × 256 pixels and using the Box-Cox transformation technique for normalization. Furthermore, the proposed model incorporates Depthwise separable convolution (DSC) blocks, which extract fine-grained features from both modalities at the pixel level. We further generated synthetic ultrasound images using the StyleGAN3 model, balancing the datasets due to the smaller size of the USI dataset. Even after the above steps, minor variations in datasets may have a biasing effect on the performance of the models.

Moreover, our proposed model still has some limitations that we will try to improve in future works: it has not been tested on real-world clinical datasets yet; its optimization for mobile devices is in process. The model also shows a slight deterioration in performance in real datasets without augmentation due to data scarcity class imbalance and variations in the quality of the images, which further signifies the need for adequate and balanced training data. Future research needs to be directed towards standardized data collection and preparation protocols, validation on more diverse and real-world datasets, and increasing its deployability. It will help to make sure that the proposed approach is robust, generalizable, and clinically applicable to a wide range of real-world scenarios.

## 6. Conclusions

In this work, we have presented the novel LightweightUNet deep learning model for the effective classification of breast cancer. This model comprises only nine layers, utilizing Depthwise separable convolution blocks, which make it fast, accurate, and computationally efficient. Due to its simplified structure and low computational cost, it can be deployed on portable devices with limited resources. We have utilized a multimodal dataset, which includes ultrasound images (USI) and mammograms (MGI) from seven sources: BrEaST, BUSI, Thammasat, HMSS, DDSM, MIAS, and INbreast.

The original (actual) dataset contains 13,000 images, including 10,000 MGI and 3000 MGI, among which 10,400 images were used to train the proposed model using a 5-fold cross-validation technique. On the real dataset, the model produced good results (86.87% accuracy, 86.84% F1-score, 86.87% recall, and 87.16% precision); no data augmentation was used.

To enhance the model performance, we generated synthetic USI images to balance the dataset, as the number of ultrasound images is smaller than that of mammogram images. By utilizing the StyleGAN3 model, we generated 10,000 synthetic USI images, significantly increasing the training dataset to 20,400 images. Outstanding results were obtained on the combined (Real + GAN) dataset, achieving 96.36% precision, 96.35% recall, 96.35% F1-score, and 96.35% accuracy using the multimodal technique in conjunction with data augmentation. This notable enhancement demonstrated the significant impact of the synthetic dataset generated using GAN on boosting the model’s performance.

Even while LightweightUNet produced encouraging findings, its shortcomings can be addressed in future studies to ensure its practicality. Improving the model’s applicability to a wider range of disease categories involves testing it on datasets that are both geographically and even more varied. Furthermore, researching methods to clarify the model’s decision-making procedure would improve interpretability and trustworthiness in medical contexts. Moreover, the model’s accessibility in resource-constrained contexts may be increased by evaluating and optimizing it for deployment on mobile devices. In the end, actual clinical studies are necessary to evaluate the model’s safety and efficacy in real-world healthcare environments. LightweightUNet has the potential to develop into a useful tool for accurate breast cancer diagnosis in clinical practice by addressing these future goals.

## Figures and Tables

**Table 1:** A Survey of recent research in breast cancer detection using ML/DL: Techniques, findings, and limitations.

Literature	Year	Techniques	Findings	Limitations
Khuriwal et al. [14]	2018	Median filter + CNN	Accuracy: 98.00%	Needs validation of larger datasets, high computational complexity
Valvano et al. [15]	2019	CNN	Accuracy: 98.20%	Limited feature diversity, lower generalizability
Ramadan [16]	2020	CNN	Accuracy: 92.10%	Lack of feature diversity and interpretability
Sharma and Mehra [17]	2020	Feature extraction + SVM	Accuracy: 93.90%	Feature redundancy might affect interpretability
Ur Rehman et al. [18]	2021	FC-DSCNN	Accuracy: 90.00%	Limited dataset diversity for generalizability
Sannasi Chakravarthy and Rajaguru [19]	2022	ICS-ELM	Accuracy: 98.27%	Limited explanation of incremental learning
Zhao et al. [20]	2022	YOLOv3	Accuracy: 98.09%	Lacks evaluation of external datasets
Huynh et al. [21]	2023	ROI Detection and YOLOX	Accuracy: 92.00%	High computational complexity needs generalizability investigation
Zakareya et al. [22]	2023	VGG-16	Accuracy: 95.00%	Limited interpretability exploration, lacks diverse dataset evaluation
Aidossov et al. [23]	2023	Thermal features + ResNet50	Accuracy: 90.74%	Limited data availability, lacks comparison with traditional modalities
Sheeba et al. [24]	2023	Active contour convolution neural networks	Accuracy: 97.00%	Needs external validation, limited explanation of memory mechanisms
Bouzar-Benlabiod et al. [25]	2023	GLCM features + SE-ResNet-101	Accuracy: 86.71%	Reliance on texture features might limit the detection of subtle abnormalities.
Asadi and Memon [26]	2023	U-Net	Accuracy: 98.61%	Lacks evaluation on external datasets, limited interpretability exploration
Sait and Nagaraj [27]	2024	EfficientNet B7 + LightGBM	Accuracy: 99.4%	Interpretability challenges due to model complexity
Sahu et al. [28]	2024	Multiple deep learning architectures (ResNet18, MobileNetV2, AlexNet)	Accuracy: 96.92% to 99.17%	Lacks evaluation on external datasets, limited interpretability exploration
AlSalman et al. [29]	2024	Statistical features + Federated Learning + DCNN	Accuracy: 98.9%	Scalability and privacy concerns reliance on statistical features might limit performance.
Oyelade et al. [30]	2024	TwinCNN (Histology + Mammography)	Accuracies for each modality (97.7%, 91.3%), lower for fused images (68.4%)	Challenges in effectively leveraging information from different modalities, limitations of statistical features
Zarif et al. [31]	2024	CNN + EfficientNetV2B3	Accuracy: 96.3%	Lacks evaluation on external datasets, limited interpretability exploration
Laxmisagar et al. [32]	2022	MobileNet CNN + Categorical Cross-Entropy (Lightweight DNN)	Accuracy: 87.5% in breast cancer classification of histopathological images.	Requires high-performance computing resources like powerful GPUs and cloud services (e.g., Google Colab).
Nneji et al. [33]	2023	Lightweight Separable Convolution (LWSC)	(97.23%), sensitivity (97.71%), specificity (97.93%), accuracy; Enhanced trainable features	It needs validation on diverse datasets, but it has limited comparison with state-of-the-art models.
Yan et al. [34]	2023	PLA (Privacy-Embedded Lightweight and Efficient Automated) Framework	Accuracy (95.3%), recall rate (99.8%), F1 score (96.9%), and precision (98.8%), including privacy-preserving technique.	This needs further validation on real-world IoMT deployment scenarios, as well as potential trade-offs in model complexity and privacy-preservation overhead.
Kausar et al. [35]	2023	Wavelet Transform (WT) + Lightweight CNN with Invertible Residual Block	Accuracy: 96.25% (ICIAR 2018), 99.8% (BreakHis), and 72.2% (Bracs).	Accuracy is dataset-dependent, with a noticeable performance drop on the Bracs dataset; further validation is needed for diverse datasets and real-world applications.
Oladimeji et al. [36]	2023	Lightweight deep learning + Slice Selection (entropy, variance, gradient magnitude)	Entropy-based slice selection achieved an accuracy of 91%, reducing computational cost and complexity.	It needs further evaluation of large and diverse datasets; generalizing to other imaging modalities may have limitations.
Elaraby et al. [37]	2024	Lightweight CNN (LWCNN) + Screening Mammograms	Achieved training and testing accuracy (95–99%) on two cases of original and enhancement datasets.	It requires validation of a broader range of datasets and limited analysis of false positives and false negatives.
Saha et al. [38]	2024	Breast-NET + Transfer Learning (Lightweight DCNN)	Accuracy: 90.34%, Precision: 0.86, Recall: 0.92, F1: 0.89. Used BreakHis, IDC grading, and IDC datasets.	Validation is limited to specific datasets and resource constraints in deploying models in real-world remote areas.
Chi et al. [39]	2024	ResNet34 + Chi-Square Filter + SVM (Lightweight Technique)	Achieved 99.62% accuracy for breast cancer detection from thermography images. Demonstrated high performance and improvement (18.3%) over standard methods.	It may require computational resources for training, and generalizability to other datasets may need further evaluation.
Tang et al. [40]	2025	Multi-light Net (multi-input lightweight CNN) + Weighted Label Smoothing Regularization (WLSR)	Combining front and side thermal images improves detection accuracy; WLSR enhances generalization, stability, and convergence.	Limited testing on resource-constrained mobile devices; further validation with diverse datasets is needed.

Abbreviations: ICS-ELM: Improved Crow-Search Optimized Extreme Learning Machine; GLCM: Gray-Level Co-Occurrence Matrix; ROI, region of interest; DCNN: Deep convolutional neural network; DDSM: Digital database for screening mammography; DNNS: Deep neural network with support value; FC-DSCNN: Fully connected depth wise-separable convolutional neural network; SVM: Support vector machine; YOLO: You Only Look Once.

**Table 2:** Distribution of datasets for MGI and USI by pathology type (benign and malignant) used in breast cancer detection.

Modalities	Mammogram Images (MGI)			Ultrasound Images (USI)			
Datasets	DDSM	MIAS	INbreast	BrEaST	BUSI	Thammasat	HMSS
No. of Images	10,239	322	410	256	780	263	2006
Selected Images	Benign = 5000, Malignant = 5000			Benign = 1500, Malignant = 1500			
Total	10,000			3000			

**Table 3:** Dataset distribution (Train/Test) of USI and MGI pre- and post-StyleGAN3 augmentation (Real vs. Real + GAN).

Real Dataset (Before Augmentation)				Real + GAN Dataset (After Augmentation)		
Modality	USI	MGI	Total	USI	MGI	Total
Test	600	2000	2600	600	2000	2,600
Train	2400	8000	10,400	12,400	8000	20,400

**Table 4:** LightweightUNet hyperparameter comparison across datasets.

Experimentation	Real Dataset	Real + GAN Dataset
Input Size ^a^	256 × 256	256 × 256
Training Type	5-fold cross-validation	5-fold cross-validation
Kernel Size ^b^	3	3
Number of Classes	2	2
Learning Rate ^c^	0.00001	0.00001
Optimizer ^d^	Adam	Adam
Batch Size ^e^	16	32
Number of Epochs	50	50

^a^ Each input image from both modalities has been preprocessed to a standard size of 256 × 256 pixels in RGB format. ^b^ Kernel size, which specifies the dimensions of the convolutional kernel employed in the Depthwise separable convolution blocks, is uniformly set to 5 for both experiments. ^c^ Learning rate, which governs the step size during optimization, is fixed at 1 × 10^−5^ (0.00001) to ensure gradual parameter updates during training. ^d^ Adam optimizer was utilized for conducting both experiments, which is a proven and efficient method for optimizing the parameters of the neural network. ^e^ Batch sizes vary between experiments due to differences in dataset sizes. With the real dataset being smaller compared to the Real + GAN dataset, batch sizes of 16 and 32 were optimized during experimentation, respectively.

**Table 5:** Fold-wise performance comparison of breast cancer classification using the proposed LightweightUNet model on a real dataset.

Folds	Types	Pre	Rec	F1	Acc
fold 1	Benign	88.924	76	81.955	83.267
	Malignant	79.045	90.533	84.400	
Average		83.9845	83.2665	83.1775	
fold 2	Benign	86.898	86.667	86.782	86.8
	Malignant	86.702	86.933	86.818	
Average		86.8	86.8	86.800	
fold 3	Benign	91.265	80.8	85.714	86.533
	Malignant	82.775	92.267	87.264	
Average		87.02	86.5335	86.489	
fold 4	Benign	90.054	89.333	89.692	89.733
	Malignant	89.418	90.133	89.774	
Average		89.736	89.733	89.733	
fold 5	Benign	85.185	92	88.462	88
	Malignant	91.304	84	87.500	
Average		88.2445	88	87.981	
Average	Benign	88.4652	84.96	86.521	86.867
	Malignant	85.8488	88.7732	87.1512	
	Overall	87.157	86.8666	86.8361	

**Table 6:** Fold-wise performance comparison of breast cancer classification using the proposed LightweightUNet on real + GAN.

Folds	Types	Pre	Rec	F1	Acc
fold 1	Benign	92.945	95.733	94.319	94.233
	Malignant	95.601	92.733	94.146	
Average		94.273	94.233	94.2325	
fold 2	Benign	95.652	96.8	96.223	96.2
	Malignant	96.761	95.6	96.177	
Average		96.2065	96.2	96.2	
fold 3	Benign	95.976	97	96.485	96.467
	Malignant	96.968	95.933	96.448	
Average		96.472	96.4665	96.4665	
fold 4	Benign	97.642	96.6	97.118	97.133
	Malignant	96.636	97.667	97.149	
Average		97.139	97.1335	97.1335	
fold 5	Benign	97.732	97.667	97.699	97.7
	Malignant	97.668	97.733	97.701	
Average		97.7	97.7	97.7	
Average	Benign	95.9894	96.76	96.3688	96.347
	Malignant	96.7268	95.9332	96.3242	
	Overall	96.3581	96.3466	96.3465	

**Table 7:** The computational complexity of the proposed LightweightUNet model utilizing GPU and CPU processors in the training and testing phase.

Computational Complexity	Image Size	GPU (50 Epochs, 5-Fold CV)	CPU (50 Epochs, 5-Fold CV)
Training Time	256 × 256	91 min	162 min
Testing Time	256 × 256	0.00392 min	0.00392 min

## Data Availability

This study uses a publicly accessible dataset that can be accessed here: INbreast: https://www.kaggle.com/datasets/ramanathansp20/inbreast-dataset accessed on 15 May 2024; HMSS: https://www.ultrasoundcases.info/ accessed on 1 May 2024; DDSM: http://www.eng.usf.edu/cvprg/mammography/database.html accessed on 15 May 2024; Thammasat: http://www.onlinemedicalimages.com/index.php/en/81-site-info/73-introduction accessed on 15 May 2024; BUSI: https://scholar.cu.edu.eg/?q=afahmy/pages/dataset accessed on 15 May 2024; BrEaST: https://www.cancerimagingarchive.net/collection/breast-lesions-usg/ accessed on 15 May 2024; MIAS: https://www.repository.cam.ac.uk/items/b6a97f0c-3b9b-40ad-8f18-3d121eef1459 accessed on 15 May 2024.
